# Genome-wide epigenetic and proteomic analysis reveals altered Notch signaling in EPC dysfunction

**DOI:** 10.14814/phy2.12358

**Published:** 2015-04-28

**Authors:** Jamie R Karcher, Brian R Hoffmann, Pengyuan Liu, Yong Liu, Mingyu Liang, Andrew S Greene

**Affiliations:** 1 Biotechnology and Bioengineering Center, Medical College of WisconsinMilwaukee, Wisconsin; 2 Department of Physiology, Medical College of WisconsinMilwaukee, Wisconsin; 3 Cancer Center, Medical College of WisconsinMilwaukee, Wisconsin

**Keywords:** Angiogenesis, endothelial progenitor cells, epigenetics, high-salt diet, Notch4

## Abstract

Endothelial progenitor cells (EPCs) are bone-marrow-derived mononuclear cells that participate in tube formation in vitro and vessel formation in vivo. EPC transplantation, as a therapeutic approach in cardiovascular diseases, has produced mixed results likely due to underlying disease states and environmental factors affecting EPC function. In this study, we investigated the mechanisms by which a high-salt diet impairs EPC function. The number of endothelial progenitor cells (CD34^+^, VEGFR2^+^, CD133^+^, and c-Kit^+^) was decreased in the bone marrow of Sprague–Dawley (SD) rats fed a high-salt diet (HSD; 4% NaCl) as compared to SD rats on a normal-salt diet (NSD; 0.4% NaCl). NSD EPCs augmented endothelial cell tube formation in vitro, whereas HSD EPCs did not. NSD EPCs were a potent therapeutic restoring electrical stimulation-induced angiogenesis in vivo. HSD EPCs were not able to restore angiogenesis in vivo. EPC DNA methylation was analyzed by reduced representative bisulfite sequencing and membrane proteins were analyzed using high accuracy liquid chromatography mass spectrometry. Differentially methylated genes and differentially abundant membrane proteins measured between the NSD and HSD EPCs, revealed a total of 886 gene-protein sets where reciprocal methylation and expression occurred. Based on stringent criteria, Notch4 was found to be hypermethylated in HSD EPCs and had corresponding decrease in protein expression. Suppression of Notch4 protein expression in EPCs using siRNA confirmed a role for Notch4 in EPC-mediated angiogenesis, suggesting Notch4 suppression as a mechanism by which high-salt diet inhibits EPC-mediated angiogenesis.

## Introduction

Endothelial progenitor cells (EPCs) are bone-marrow-derived cells that exhibit endothelial cell properties in vitro and promote angiogenesis in vivo (Asahara et al. 1997). EPCs make up approximately 1% of the total bone marrow mononuclear cell population (Asahara et al. 1997). A wide variety of cellular markers and growth conditions have been used to define the EPC population. In this study, EPCs were defined as the population of cells that adhere to fibronectin-coated plates and are positive for CD34, VEGF receptor 2 (VEGFR2), c-Kit, and CD133 after 14 days in culture in EPC-specific media.

Experimental studies and some preclinical trials have shown that autologous injection of EPCs during ischemic tissue conditions can increase capillary density (Orlic et al. 2001; Murphy et al. 2007). EPC treatment may improve cardiac function in myocardial infarction models (Kawamoto et al. 2001), as well as in a model of hypertensive heart failure (Parker and Greene 2011). EPC transplantation also increased microvessel density and decreased fibrosis in cardiac tissue in a salt-sensitive hypertensive rat model, by increasing blood flow and an improving diastolic function (Parker et al. 2012). Under some experimental conditions EPCs can incorporate into blood vessels (Yeh et al. 2003; Ziegelhoeffer et al. 2004; O'Neill et al. 2005; Peters et al. 2005), however, it appears that the rate of vascular incorporation is generally very low.

Many of the benefits found with the EPC therapy in experimental animals have not been replicated in human clinical trials (Kuethe et al. 2004; Lunde et al. 2005). A number of explanations for the varying results found in human EPC trials have been offered, however, the most likely explanation is that the source of EPCs in animal studies were from healthy animals with experimentally induced cardiovascular injury or disease, whereas in human trials EPC transplants were autologous and potentially dysfunctional.

Dysfunction of EPCs has now been demonstrated in diseases including diabetes, atherosclerosis, stroke, and hypertension (Vasa et al. 2001; Tepper et al. 2002; Loomans et al. 2004a; Pistrosch et al. 2005). When isolated from the blood of patients with congestive heart failure, EPCs show impaired migration and neovascularization capacity (Walter et al. 2005; Geft et al. 2008). Hypertension has also been correlated with decreased circulating numbers of EPCs (Fadini et al. 2006; Pirro et al. 2007), decreased migration of EPCs (Vasa et al. 2001; Imanishi et al. 2005), and decreased survival of EPCs in the blood (Imanishi et al. 2005). EPCs from hypertensive patients also display increased senescence (Yao et al. 2008; Zhou et al. 2008). The mechanisms underlying the EPC dysfunction are not clear. However, the impairment in EPC proliferation, adhesion, and angiogenic properties may underlie the failure of EPC transplants and understanding the mechanisms involved in EPC dysfunction may improve our understanding in cardiovascular disease pathogenesis and vascular complications. Development of new strategies to restore EPC function and consequently increase EPC engraftment and/or mobilization may considerably impact angiogenic stem cell-based therapy.

In this study, high-salt diet was tested as a model of EPC dysfunction. We found that a high-salt diet (4% NaCl, HSD) caused a decrease in the relative number of EPCs in the bone marrow mononuclear cell (BM-MNC) fraction but it did not alter cellular marker expression of isolated and cultured EPCs. Unlike normal-salt diet (0.4% NaCl, NSD) treated rats, HSD EPCs could not augment in vitro tube formation when cocultured with endothelial cells. In vivo transplantation of NSD EPCs caused a robust therapeutic restoration of stimulated angiogenesis, however, HSD EPCs did not. DNA methylation and proteomic analysis of NSD and HSD EPCs showed numerous gene-protein sets with altered methylation or expression. Bioinformatic analysis revealed Notch4 as a lead candidate in EPC dysfunction that was confirmed by experimental knockdown.

## Methods and Materials

### Animal care

Male Sprague–Dawley rats were purchased from Harlan Laboratories (Indianapolis, IN) and were 7–9 weeks old at the time of this study. The Medical College of Wisconsin (MCW) Institutional Animal Care and Use Committee approved all animal protocols. Animals were housed and cared for in the MCW Animal Resource Center and were given food and water ad libitum.

### Endothelial progenitor cell isolation from the bone marrow

Sprague–Dawley rats were placed on a normal-salt diet (NSD, 0.4% NaCl Dyets) or a high-salt diet (HSD, 4% NaCl Dyets) for 7 days. Following 7 days on either diet, the rats were killed with Beuthanasia-D Special (Merck) and both femurs and tibias were surgically dissected. The bone marrow mononuclear cell population was isolated as previously described (Kaczorowski et al. 2013; Karcher and Greene 2014). The bone marrow mononuclear cells were resuspended in endothelial cell basal medium-2 [EGM-2 (Lonza); MCDB131 media containing 10% heat-inactivated FBS, vascular endothelial growth factor (VEGF), human fibroblast growth factor-2, human epidermal growth factor, insulin-like growth factor-1, ascorbic acid, gentamicin, and amphotericin-B]. To isolate EPCs the bone marrow mononuclear cells were plated on bovine fibronectin (Sigma, St. Louis, MO, F1141) coated 100 mm tissue culture dishes at a concentration of 1 × 10^7^ cells/plate. The plates were incubated in 21% O_2_ and 5% CO_2_ at 37°C until the cells were approximately 70% confluent (14 days).

### Immunocytochemistry and flow cytometry

Bone marrow mononuclear cells (BM-MNCs) or endothelial progenitor cells were isolated and characterized using fluorescently labeled antibodies. The number of CD34^+^/VEGFR2^+^ cells were quantified in bone marrow mononuclear cells as previously described (Parker et al. 2012). EPCs were labeled with CD34, c-Kit, CD133, and VEGR2 to validate known cellular makers. Briefly BM-MNCs or EPCs were fixed in 2% paraformaldehyde solution in Dulbecco's Phosphate-Buffered Saline (DPBS). The cells were incubated in 90% methanol solution for 30 min and blocked in 3% goat serum. The bone marrow mononuclear cells were incubated with monoclonal antibodies against CD34 (Courtesy of Jan E. Schnitzer (Testa et al. 2009), Prism, San Diego, CA) followed by Alexa Fluor 633-conjugated secondary antibody (goat anti-mouse, Invitrogen, Carlsbad, CA) and with a monoclonal antibody against FLK-1 (Santa Cruz Biotechnology, Dallas, TX, sc-315, VEGFR2) followed by Alexa Fluor 488-conjugated secondary antibody (goat anti-rabbit, Invitrogen). EPCs were incubated with monoclonal antibodies against CD34 (Courtesy of Jan E. Schnitzer (Testa et al. 2009), Prism, San Diego, CA), monoclonal antibody against FLK-1 (Santa Cruz Biotechnology, sc-315, VEGFR2), polyclonal antibody against CD133 (Abcam, Cambridge, UK, ab19898) or the polyclonal antibody against c-kit (Santa Cruz Biotechnology, sc-168) followed by Alexa488-conjugated secondary antibody (goat anti-rabbit or goat anti-mouse, Invitrogen). Cells exposed only to the secondary antibody served as controls. Each analysis included 10–50,000 events and was run on the LSRII Flow Cytometer (Becton Dickinson, Franklin Lakes, NJ). Data were analyzed using FlowJo software (TreeStar Inc. Ashland, OR).

### Matrigel tube formation assay

Four-well chamber slides were coated with 300 *μ*L of growth factor reduced Matrigel (BD Biosciences, San Diego, CA) for 60 min at room temperature. Cultured neonatal rat cardiac microvascular endothelial cells (RMVEC, Cell Biologics) were rinsed with DPBS and lifted with trypsin. Twenty thousand RMVECs were plated on Matrigel-coated wells. EPCs from HSD or NSD donor SD rats were rinsed five times with DPBS and lifted with trypsin. Twenty thousand EPCs from each donor type were added to wells with RMVECs and total volume was brought to 1.0 mL. Cells were incubated together and two 40× magnification images of tube formation were acquired and quantified following 24 h in culture. Pipeline software (Prisco et al. 2014) was used to quantify the total length of tubes and number of segments formed.

A second set of tube formation assays were performed with EPC secretome, which is the conditioned media removed from endothelial progenitor cells in culture. After 14 days in culture EPCs were switched into fresh MCDB131 with EGM-2 supplements and 10% FBS and incubated at 37°C at either at normoxia (21% O_2_ and 5% CO_2_) or hypoxia (2% O_2_ and 5% CO_2_). EPC secretome was collected for 24 h and added to 20,000 RMVECs plated on Matrigel-coated slides. RMVECs exposed only to fresh media (no secreted proteins) served as a control. Following 24 h incubation, two 40× magnification images were acquired and quantified with Pipeline software (Prisco et al. 2014).

### Electrical stimulator surgery and EPC transplantation

Sprague–Dawley rats (7–9 weeks old) were placed on high-salt diet (4% NaCl, Dyets) 1 week before the surgery and the diet was continued for the duration of the protocol. The rats were divided into two groups: (1) received endothelial progenitor cells from Sprague–Dawley rats on a normal-salt diet, (2) received endothelial progenitor cells from Sprague–Dawley rats on a high-salt diet. Two days prior to cell injections, a battery-powered stimulator was implanted, as described previously (Linderman et al. 2000). Briefly under aseptic conditions, a subcutaneous incision was made over the thoracolumbar region and a miniature battery-powered stimulator (Linderman et al. 2000) was implanted to stimulate the hind limb musculature and secured in place. Two days after the electrical stimulator was implanted 6000 EPCs were injected intravenously into the tail vein, whereas the rats were under isoflorane anesthesia. After a total of 7 days of stimulation the rats were killed and the tibialis anterior muscle was collected for analysis.

### Analysis of vessel density

The vessel density of the harvested tibialis anterior (TA) muscle from both hind limbs of electrically stimulated rats was analyzed as previously described (Rieder et al. 1995; Kaczorowski et al. 2013; Karcher and Greene 2014; Prisco et al. 2014). Vessel density was expressed as the mean number of vessel-grid intersections per microscope field.

### RRBS library preparation and sequencing

Genomic DNA was isolated from EPCs derived from NSD and HSD fed rats with PureLink™ Genomic DNA Mini Kit (Invitrogen), following the manufacturer's recommendations. The genomic DNA was eluted in DNA elution buffer (10 mmol/L Tris-HCl (pH 9.0), 0.1 mmol/L EDTA) and the concentration was determined with a Qubit fluorometer and dsDNA BR Assay Kit per the manufacturer's instructions. The RRBS library was completed using approximately 200 ng of DNA as previously described (Gu et al. 2011; Boyle et al. 2012; Smallwood and Kelsey 2012; Liu et al. 2014). Briefly, the DNA was digested with MspI overnight at 37°C. End-repairing and A-tailing of the MspI-digested DNA fragments was prepared using polymerase-chain reaction (PCR). The end-repaired and A-tailed DNA fragments were ethanol precipitated for purification and were ligated to custom-designed adapters by PCR (Liu et al. 2014). Bisulfite conversion of size-selected DNA was performed using the QIAGEN EpiTect kit follow the manufacturer's protocol. The samples were amplified by PCR to prepare for sequencing. An Agilent Bioanalyzer DNA 1000 chip was used to validate and quantify the library. Samples were multiplexed six per lane on a 300 Gb flowcell and paired-end sequenced with an Illumina HiSeq 2000.

### Sequence alignment and analysis

Sequence reads obtained from Illumina HiSeq 2000 were analyzed by MethylCoder (Pedersen et al. 2011) using the genomic short-read-nucleotide alignment program (GSNAP) (Langmead et al. 2009; Wu and Nacu 2010). Bisulfite conversion rates were estimated from the number of unconverted cytosines at Klenow filled 3’ MspI sites of sequencing reads. The numbers of converted and unconverted cytosines across the genome were extracted from each RRBS library. For each region of DNA where a cytosine occurred next to a guanine (CpG), the amount of methylation on the 5’ position of the cytosine (5mC) was taken as the percentage of unconverted cytosines. The boundaries of CpG islands (CGIs) were downloaded from the UCSC genome annotation database. An overall value of 5mC per CGI was calculated by pooling data from all the CpGs covered within each CGI. CpGs with fewer than 20 reads were excluded, and only CGIs with five representative CpGs or more were analyzed. To test for CGIs that contained 5mC levels significantly above the bisulfite conversion error in each RRBS library, a binomial test was applied using a Benjamini–Hochberg corrected *P*-value cutoff of 0.05. Fisher's test was used to evaluate differences between groups.

### Preparation of samples for mass spectrometry

EPCs were collected from NSD and HSD fed rats. EPCs derived from each rat were rinsed twice and scraped in DPBS, pooled and pelleted at 300 ×g for 5 min at 4°C. Cells were lysed mechanically twice in 4 mL of hypotonic lysis buffer (1 mol/L Tris (pH 7.5) and 100 mmol/L MgCl_2_) by 30 passages with a cooled glass dounce. Lysate was centrifuged at 800 ×g for 10 min at 4°C to pellet nuclei and large organelles. The supernatant was transferred to a clean tube and the pellet was rinsed with a membrane preparation buffer (280 mmol/L sucrose, 50 mmol/L MES pH 6.5, 450 mmol/L NaCl, and 10 mmol/L MgCl_2_) for 10 min on ice. Supernatants were centrifuged at 75,000 ×g at 4°C overnight to pellet membranes. The pelleted membranes were washed with 200 μL of fresh membrane wash buffer (25 mmol/L NaCO_3_) and set in thermomixer at 4°C for 30 min. Membranes were spun in the ultracentrifuge at 75,000 ×g at 4°C for 30 min. Samples were reduced in 100 mmol/L Ammonium bicarbonate and RapiGest (Waters) for 20 min. Fresh 100 mmol/L Iodoacetamide was added to cause alkylation for 20 min on a thermomixer at 55 ×g for 20 min. Trypsin (one vial of Promega Sequence-grade-modified trypsin reconstituted (a total of 20 μg added to sample)) was added to the samples to digest overnight at 37°C.

The samples were spun at 14,000 ×g for 10 min to remove any undigested debris. The peptide solutions were dried in a speed vacuum and subsequently resuspended in 0.1% TFA for desalting on Varian Omix C18 desalting tips (Agilent Technologies, Santa Clara, CA). Tryptic peptide samples were resuspended in buffer A (98% HPLC H_2_O, 1.9% CAN (acetonitrile), 0.1% formic acid). Protein concentrations were measured after cell lysis and diluted appropriately to normalize protein loading. Tryptic peptide samples (1.9 *μ*L/run) were passed over an in-house C18 resin (particle size 5 *μ*m; Phenomenex, Torrance, CA) packed 15 cm column (inner diameter of 50 *μ*mol/L) coupled to a NanoAccuity UPLC system (Waters, Milford, MA). A 240-min gradient from buffer A to buffer B (98% ACN, 1.9% HPLC H2O, and 0.1% FA) was applied to the peptide-bound C18 column. Eluted peptides underwent electrospray ionization followed by data acquisition using an LTQ-Orbitrap Velos Mass Spectrometer (Thermo Scientific, Waltham, MA). Each group (NSD and HSD EPCs) had data acquired for three biological replicates. Each biological replicate included two technical replicates for a total of 12 runs MS runs.

### Proteomic data analysis

Mass Spectrometric RAW data files were uploaded into the Medical College of Wisconsin Biotechnology and Bioengineering Center Workflow and extracted using ExtractMSn 5.0, followed by Sequest and Mascot database search. Searches were performed against the UnitProtKB rodent database. Using Visualize Software (Halligan and Greene 2011), Sequest and Mascot searches for each individual run were combined via the search combiner matching each spectra to the best match from either search algorithm to avoid redundancy. Each biological replicate then had all Sequest/Mascot combined files for each technical replicate compiled, followed by compilation of all EPC replicates. A relative quantitation comparison of the total values for NSD versus HSD EPCs through spectral counting was performed and normalized for overall scan count (Halligan and Greene 2011; Parker and Greene 2011; Kaczorowski et al. 2013). Filters included removal of redundant proteins, presence in two of three biological replicates, scan count ≥ 25 for either condition, protein probability >0.85, at least two peptides identified, and at least a twofold change in protein expression.

### Immunoblot to determine Notch4 protein expression

EPCs from Sprague–Dawley rats on NSD or HSD were harvested as described above. Cells were placed in normoxic (21% O_2_, 5% CO_2_) or hypoxic conditions (2% O_2_, 5% CO_2_) at 37°C for 6 h and were flash frozen in mammalian protein extraction reagent (MPER, Thermo Fisher Scientific) containing protease inhibitors (Roche). Immunoblots were performed as previously described (Karcher and Greene 2014) using a rabbit polyclonal antibody for Notch4 [Anti-Notch4 (c-3), sc-377399, dilution 1:500]. Membranes were stained with Ponceau S (Sigma) to confirm equal protein loading. Whole kidney lysate was used as positive control.

### Quantitative PCR to detect Notch4

EPCs were harvested as described above. Cells were incubated for 6 h at normoxic (21% O_2,_ 5% CO_2_) or hypoxic conditions (2% O_2,_ 5% CO_2_) at 37°C and total RNA was purified using Qiagen's RNeasy Mini Kit per manufacturer's instructions. Real-time PCR analysis was performed as described by Knoll et al. (2005) with a Notch4 primer set (Rn01525734_g1, Thermo Scientific). Notch4 mRNA expression was normalized to endogenous 18S rRNA.

### siRNA-mediated Notch4 knockdown experiments

After 13 days of expansion, EPCs cells were transfected using DharmaFECT3 transfection reagent (T-2003-03; Thermo Scientific) with ON-TARGETplus Notch4 siRNA (J-103283-05-0005, Thermo Scientific) or the scrambled control (D-001810-02-05, Thermo Scientific) according to the manufacturer's instructions. The transfection mixture and siRNA was added to MCDB131^+^ EGM-2 (without antibiotics) media yielding a siRNA final concentration of 25 nmol/L. After 24 h of transfection, the media on the transfecting EPCs was changed to MCDB131^+^ EGM-2 (with antibiotics). After 24 h, the cells were used in the tube formation assay. RNA was isolated from an aliquot of cells was used to assess knockdown efficiency.

### Statistics

Significance was determined by one-way or repeated measures ANOVA (Sigma Plot, SPSS Inc., Chicago, IL) with post hoc Fisher's least significant difference where appropriate. All results are reported as mean ± standard error (SE). Computation of statistical significances of observed ms/ms spectra in HSD compared to NSD EPC samples were appropriately adjusted for multiple comparisons (Halligan and Greene 2011). Statistical comparisons of tube formation and cell surface marker expression were performed with t-tests. One-way analysis of variance was used to compare differences between group for histological and functional measurements from the in vivo experiments. Student Neuman–Keuls post hoc testing was performed when significant group differences were indicated. Significance was set at *P* < 0.05.

## Results

### Quantification of endothelial progenitor cells

**Figure 1:**
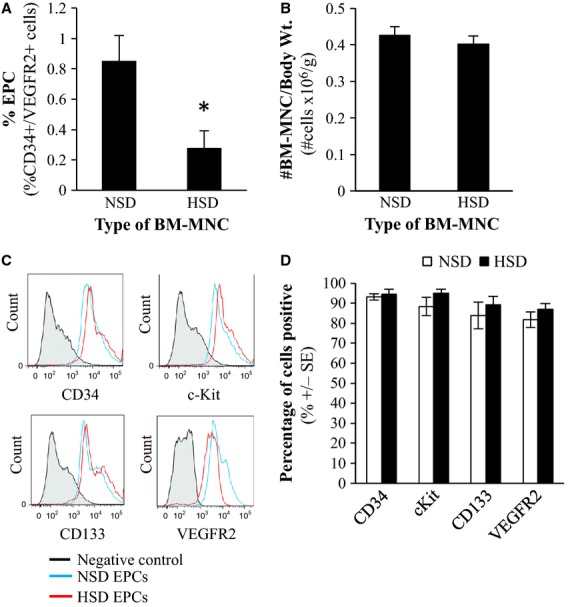
(A) Quantification of number of endothelial progenitor cells (EPCs) in the bone marrow mononuclear cell fraction. Percent of EPCs expressed as number of CD34 positive and VEGFR2-positive cells in the total bone marrow mononuclear cell population. *Significantly different than the normal-salt diet (NSD) group. (B) Total number of BM-MNCs isolated from donor rats with respect to the final body weight of the donor rat. (C) Representative histograms depicting the flow cytometry results for each group. Negative control was EPCs stained only with secondary antibody. (D) The number of cells positive for each marker was quantified for NSD EPCs and HSD EPCs. Graphical representation of flow cytometry data. NSD = normal-salt diet, HSD = high-salt diet. Data expressed as mean ± SE (*n* = 3–4).

HSD led to a significant decrease in the number of CD34^+^/VEGFR2^+^ cells relative to normal-salt fed rats (*P* = 0.029), suggesting that HSD significantly decreased the number of EPCs in bone marrow available for self-repair (Fig.1A). The total number of mononuclear cells isolated from the bone marrow between the normal-salt and high-salt donors did not differ (Fig.1B).

### Cell surface marker expression of enriched EPCs

Expression of the phenotype markers VEGFR2, c-Kit, CD133, and CD34 on cultured EPCs derived from high-salt and normal-salt fed donor rats is shown in Fig.1C and D. Cell marker composition of EPCs isolated from high-salt donors did not differ from EPCs isolated from normal-salt diet fed Sprague–Dawley donor rats.

### EPC tube formation

**Figure 2:**
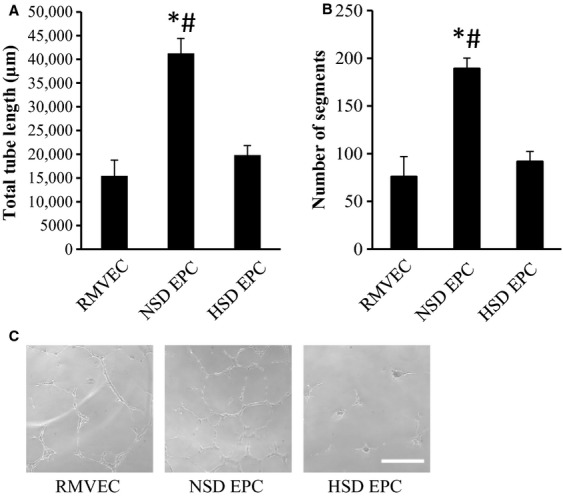
EPC tube formation assay. Endothelial progenitor cells isolated from NSD (*n* = 4) or HSD (*n* = 5) donor rats were coincubated with rat microvessel endothelial cells (RMVECs) on Matrigel. (A) Total tube length after 24-h incubation. (B) Number of segments after 24-h incubation. Data expressed as mean ± SE. *Significantly different than RMVEC. ^#^Significantly different than HSD EPC (*P* ≤ 0.05). (C) Representative images 10× from RMVEC only, NSD EPC and HSD EPC tube formation, bar = 500 *μ*m.

The angiogenic capacity of NSD and HSD endothelial progenitor cells was analyzed using an in vitro tube formation assay. HSD EPCs cocultured with rat heart microvessel endothelial cells (RMVECs) formed fewer tubes than NSD EPCs cocultured with RMVECs as quantified by total tube length and number of segments. (Fig.2).

### Effect of exogenous EPC delivery on microvessel density in nonhypertensive SD recipients on a high-salt diet

**Figure 3:**
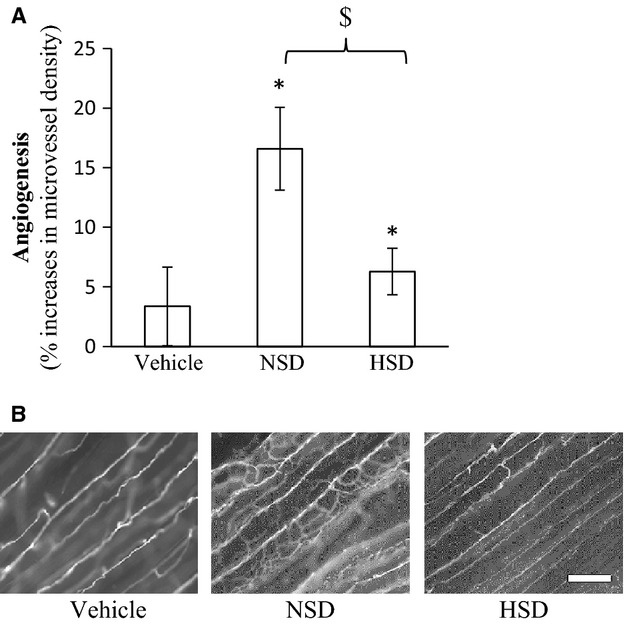
EPC transplantation with electrically stimulated angiogenesis. Vehicle or 6000 EPCs from NSD or HSD donor rats were injected intravenously through the tail vein of HSD SD donor rats receiving electrical stimulation. Angiogenesis was determined in the tibialis anterior following EPC transplantation. Data expressed as mean ± SE. *Significantly increased in microvessel density between the stimulated and unstimulated limb. ^$^Significantly different between NSD and HSD (*P* ≤ 0.05, *n* = 9). (B) Representative images of stimulated muscle, bar = 50 *μ*m.

Following intravenous injection of 6000 EPCs, there was a significant difference between NSD and HSD EPC transplantation where HSD EPCs were significantly less able to facilitate electrically stimulated angiogenesis than NSD EPCs (Fig.3).

### EPC secretome tube formation

**Figure 4:**
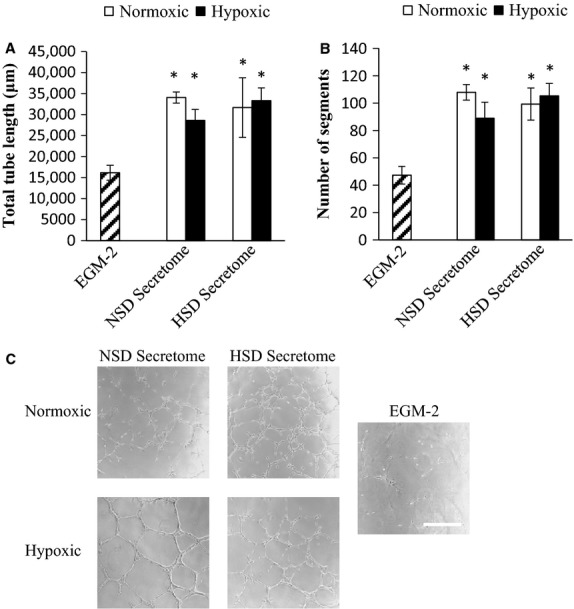
EPC secretome tube formation assay. Secretome was collected from NSD and HSD EPCs by exposing the cells for 24 h to normoxic (21% O_2_, 5% CO_2_) or hypoxic (2% O_2_, 5% CO_2_) environment. The secretome was added to 20,000 rat microvascular endothelial cells (RMVEC) seeded on Matrigel-coated chamber slides. Control sample was RMVECs exposed to fresh media (EGM-2) only. (A) Total tube length after 24-h incubation. (B) Number of branch points after 24-h incubation. Data expressed as mean ± SE. *Significantly different from control (EGM-2) group (*P* ≤ 0.05, *n* = 3). (C) Representative images 10× from EGM-2 only, secretome from NSD and HSD EPCs collected following normoxic or hypoxic treatment, bar = 500 *μ*m.

The angiogenic capacity of NSD and HSD secretome was analyzed using an in vitro tube formation assay. The secretome was collected following incubation in a normoxic (21% O_2_ and 5% CO_2_) or hypoxic (2% O_2_ and 5% CO_2_) environment for 24 h. Both NSD- and HSD EPC-derived secretome were highly angiogenic (Fig.4). Neither the diet of the donor nor the exposure of the EPCs had any effect on the angiogenic potency of EPC secretome.

### DNA methylation

Three NSD and three HSD EPC samples, each derived from a separate biological experiment, were prepared, bisulfite converted and sequenced. The number of reads (51–53 million, *P* = 0.464), mapping rate (approximately 79.3 ± 0.4%, *P* = 0.700), bisulfite conversion efficiency (99.5 ± 0.04%, *P* = 0.725), and methylation rate (approximately 26.7 ± 0.6%, *P* = 0.817) were similar between all samples. The number of significantly methylated CpG islands (CGI) in NSD and HSD EPC samples was similar (average 6640 ± 35 CGIs). The average methylation rate in significantly methylated CGIs differed by genomic location but was similar between NSD and HSD EPCs. CGIs were categorized into three different types: transcription start site (TSS), intragenic, and intergenic. TSS CGIs were classified as CGIs that occurred within 1000 base pairs upstream and downstream of a transcription start site. Intragenic CGIs were located within genes and intergenic CGIs occur between genes. There was a significantly greater level of methylation in intragenic sites as compared to TSS and intergenic sites. Following filtering the data for significantly different methylation levels (*P* ≤ 0.05) and filtering for false discovery rate (FDR ≤ 0.05), 150 differentially methylated genes were identified. Eighty-seven genes had significantly increased methylation in HSD EPCs as compared to NSD EPCs. Sixty-three genes had significantly increased methylation in NSD EPCs. The full list of significantly different methylated genes is in Table S1.

### Membrane protein expression for NSD and HSD EPCs

There were a total of 4114 proteins detected in the membrane fraction NSD and HSD EPC samples after combining the biological and technical replicates. Following filtering by setting minimum limits on scan count (≥25 scans) and peptide (≥2 unique peptides), a twofold change in expression and a *P*-value less than 3.0 × 10^−6^, 263 candidate proteins were identified. Fifty-one proteins were significantly increased in HSD EPCs, 60 proteins were significantly increased in NSD, 78 proteins were unique to HSD EPCs, and 74 unique to NSD EPCs. The full list of significantly differentially expressed membrane proteins meeting these criteria is listed in Table S2.

Of the significantly different proteins detected, 65.7% were canonically annotated to the plasma membrane in the NSD EPCs samples and 67.2% were canonically annotated to the plasma membrane in the HSD EPCs samples demonstrating enrichment of plasma membrane proteins using this technique.

### Correlated DNA methylation rates with protein expression

To test the hypothesis that impaired EPC function was mediated by differentially methylated and subsequent regulation of membrane proteome, we compared all genes found in the RRBS data with all of the proteins found through the proteomic analysis. We identified 886 gene-protein sets with both significantly different (*P*-value less than 0.05) DNA methylation and significantly different protein expression between the NSD and the HSD samples. Of these, 217 sets had increased HSD EPC methylation and decreased HSD EPC protein expression. One hundred and seventy-three sets had decreased HSD EPC methylation and increased HSD EPC protein expression. There were also 496 sets of gene-protein data that did not have reciprocal expression between DNA methylation and protein expression. This list of 496 sets included gene-protein sets that had increased HSD methylation and increased HSD protein expression and it included sets with decreased HSD methylation and decreased HSD protein expression.

We further narrowed down the list of potential gene-protein sets to include only gene-protein sets with reciprocal expression. These sets were analyzed further by setting limits for the *P*-value, fold change and false discovery rate (FDR) values. To be included the gene-protein list required a DNA methylation *P*-value less than 0.005 and a FDR less than 0.02. Also the gene-protein set required at least a twofold change in protein expression with a *P*-value less than 0.002. This produced a list of 129 gene-protein sets where 89 sets had increased HSD DNA methylation and decreased HSD protein expression and 40 sets had decreased HSD DNA methylation and increased HSD protein expression. Informatic analysis including gene ontology enrichment of this list identified Notch4 as a lead candidate that had a 2.4-fold increase in HSD methylation in the transcription start site and the protein product of which was found only in the NSD EPC samples. The list of all the 129 gene-protein sets can be found in Table S3.

### Notch4 expression and knockdown results

**Figure 5:**
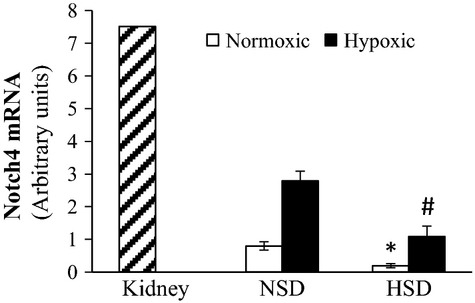
mRNA expression of Notch4 in kidney homogenate (positive control) and EPCs treated with normoxic (21% O_2_, 5% CO_2_) or hypoxic (2% O_2_, 5% CO_2_) conditions from NSD or HSD donor rats (*n* = 3). Data shown as mean ± SE. Data normalized to 18S expression. *Significantly different than NSD EPCs normoxic. ^#^Significantly different than NSD EPC hypoxic.

We examined the Notch4 mRNA expression by quantitative real-time PCR. Cells were treated with either normoxic (21% O_2_, 5% CO_2_) or hypoxic (2% O_2_, 5% CO_2_) incubation at 37°C for 6 h in order to mimic the effects of the ischemia seen in vivo. HSD EPCs had significantly lower Notch4 mRNA expression following treatment with normoxia and hypoxia as compared to NSD EPCs (Fig.5).

**Figure 6:**
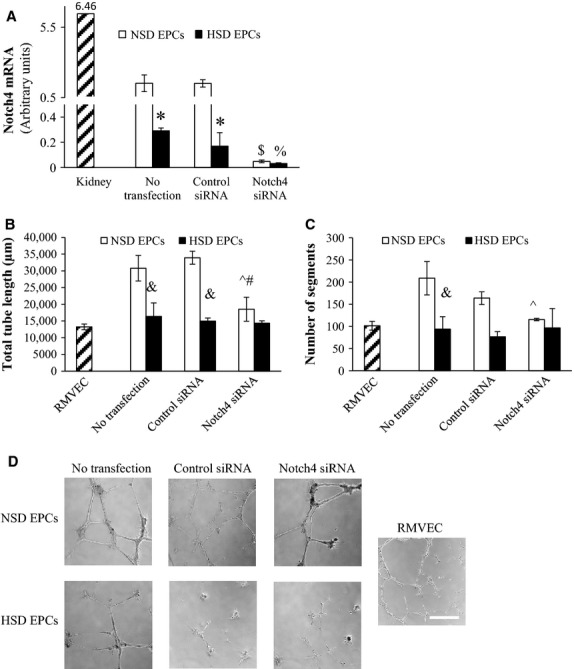
(A) mRNA expression of Notch4 in kidney homogenate and EPCs treated with no transfection, control siRNA, or Notch4 siRNA *Significantly different than NSD EPCs from same treatment. ^$^Significantly different than NSD EPCs treated with no transfection and NSD treated with control siRNA. % Significantly different than HSD EPCs treated with no transfection and HSD treated with control siRNA. (B) EPC tube formation assay. Endothelial progenitor cells were coincubated with rat microvessel endothelial cells (RMVEC) on Matrigel. Total tube length and number of segments was quantified. Total tube length after 24-h incubation. (C) Number of segments after 24-h incubation. Data expressed as mean ± SE. & Significantly different than NSD EPCs in same treatment. ^#^Significantly different than NSD EPCs treated with control siRNA. ^Significantly different than NSD EPCs no transfection (*P* ≤ 0.05, *n* = 3). (D) Representative images for RMVEC only, no-transfected, control siRNA and Notch4 siRNA-treated NSD and HSD EPCs, bar = 500 *μ*m.

Inhibition of Notch4 with Notch4-specific siRNA caused a significant decrease in Notch4 mRNA expression in NSD EPCs (Fig.6A). Inhibition of Notch4 also suppressed NSD EPC mediated tube formation by suppression of the total tube length formed (Fig.6B) and the number of segments of tubes formed (Fig.6C).

## Discussion

Endothelial progenitor cells are believed to contribute to vascular regeneration and repair processes (Alev et al. 2011). However, the presence of certain diseases appears to reduce the number of these cells in the bone marrow and diminish the function of the cells remaining (Vasa et al. 2001; Rauscher et al. 2003; Loomans et al. 2004a; Dimmeler and Leri 2008). In this study, high-salt diet decreased both the number and impaired the function of endothelial progenitor cells derived from bone marrow. A similar reduction in the number of EPCs has been observed in the Dahl salt-sensitive rat (SS/MCWi), another model of low renin activity (Parker et al. 2012) and impaired endothelial cell function. In that study reduced numbers of EPCs were hypothesized to impair the regenerative potential of bone marrow in a model of heart failure. Several studies have demonstrated disease-based suppression of EPC angiogenic capacity, proliferation and overall number in myocardial infarction, hypertension, and other cardiovascular diseases (Tepper et al. 2002; Loomans et al. 2004a,b; Pistrosch et al. 2005). To our knowledge this is the first description of an environmental factor, high-salt diet, impacting EPC number and function.

In this study EPCs were isolated using a 14-day cell culture protocol where bone marrow mononuclear cells freshly harvested from a donor rat were cultured on fibronectin-coated plates in EPC-specific media. This isolation procedure was validated by analyzing known cellular markers of EPCs including CD34, VEGFR2, c-Kit, and CD133. There was no difference in the expression of these markers between NSD- and HSD-derived EPCs suggesting that the population of cells isolated using this procedure was not altered by dietary salt.

The ability of EPCs to augment in vitro angiogenesis was tested using a Matrigel-based tube formation assay. NSD EPCs significantly increased endothelial cell tube formation as compared to endothelial cell only controls, whereas HSD EPCs were unable to augment endothelial cell tube formation. This suggests that HSD not only alters the relative number of EPCs in the bone marrow mononuclear cell fraction, but it also causes a functional defect in these cells that is maintained through 14 days in culture. Endothelial progenitor cell dysfunction has been described as changes in the following: migration, adhesion, angiogenesis, and proliferation. We chose to only look at one aspect of EPC dysfunction, angiogenesis because we believe the angiogenic potential of endothelial progenitor cells in a clinically relevant function of these cells. Future studies looking at migration, adhesion, and proliferation of EPCs isolated from high-dietary salt fed rats would lead us to better understand other areas of EPC dysfunction.

HSD EPCs were also found to have decreased ability to restore in vivo angiogenesis following intravenous transplantation. NSD EPCs were capable of restoring angiogenesis when only 6000 cells were intravenously injected into the rat. When 6000 HSD EPCs were transplanted significantly less augmentation of angiogenesis occurred.

A key finding of this work was that the effect of dietary salt persisted through 14 days of cell culture. Once isolated, all cells underwent the same culture and purification protocol with the same conditions, demonstrating that these cells retain a “memory” of their dietary salt in vivo. During the 14-day cell culture preparation of the EPCs, the cells went through many rounds of doubling suggesting epigenetic changes in vivo impacted the cellular phenotype in vitro.

DNA methylation plays an important role in mammalian development and is frequently altered in diseases, including cancer (Das and Singal 2004). Different types of cells have been shown to have different levels of methylation. For example, embryonic stem cells typically have a methylation rate of 10–15%, whereas cancer cells have methylation rates near 50% (Bird 2002). Fully differentiated cells may have methylation rates of CpG islands as high as 80% (Bird 2002). In this study samples derived from NSD and HSD EPCs had the same global rate of methylation and overall number of methylated regions. Differences in methylation were confined to individual genes where the methylation rates of CpG islands in transcription start sites, intragenic, and intergenic regions varied between NSD and HSD EPCs. To our knowledge this is the first study examining genome-wide methylation changes in EPCs in response to an environmental stimulus.

To exert their therapeutic effect following intravenous delivery, EPCs must survive in the circulation, travel to sites of inflammation and/or ischemia, recognize key signals at those sites promoting their homing, bind to the endothelium, undergo transendothelial migration, and localize to the site of tissue damage. If the incompetence of HSD-derived EPCs is caused by impairment in one of these functions, the abundance of a protein or proteins on the cell surface may be altered. Hypoxia was used as a stimulus to assess the competence of EPCs following exposure to HSD. Although the exposure to hypoxia was prolonged, 6 h or 24 h, we found no difference in secretome tube formation or Notch4 expression following between normoxic and hypoxic-treated samples suggesting that a strong hypoxic stimulus is capable of overcoming the physiological differences in EPC function observed in vivo.

To define the subset of genes where methylation might directly impact EPC function, we examined the membrane proteome based on the hypothesis that the impaired EPC function was likely do to altered cell–cell interactions in vivo. Focus on the membrane proteome was warranted because secreted proteins from NSD and HSD EPCs acted similar to augment in vitro tube formation (Fig.4). The membrane fraction of EPCs is likely to contain key proteins involved in cell migration, cell–cell, and cell-matrix interaction that are key mediators of these EPC functions.

Increases in DNA methylation are generally thought to reduce protein expression by chromatin remodeling. Cells use DNA methylation to suppress gene expression. Early reports by McGhee and Ginder in 1979 compared the methylation of the beta-globin gene (McGhee and Ginder 1979). They found cells that expressed the beta-globulin gene were unmethylated, whereas cells that did not express the beta-globulin were methylated (McGhee and Ginder 1979). This evidence was one of the first suggestions that methylation was involved in gene expression. Methylation is believed to play a crucial role in repressing gene expression by potentially blocking binding sites for transcription factors at gene promoters (Jones 2012). DNA methylation is also essential for embryonic development and cell differentiation. Methylation near gene promoters varies considerably depending on cell type, with increased methylation of promoters correlating with low or no transcription (Suzuki and Bird 2008). However, it is important to note that the function of DNA methylation varies with the relationship between the location of the DNA methylation site and other epigenetic marks on the DNA and surrounding histones (Jones 2012). The relationship between changes of DNA methylation and gene expression may be more complex when the changes are induced by physiological stimuli compared to developmentally determined changes (Liu et al. 2014).

Neurogenic locus notch homolog protein 4 (Notch4) was found to have increased methylation in HSD EPCs and had suppressed protein expression in HSD EPCs. The methylation site found in our analysis for Notch4 was located at the transcription start site of Notch4. Methylation near the transcription start site is thought to cause reduced transcription of that gene. In this study we consistently found Notch4 to be hypermethylated (2.4-fold) in three HSD EPC samples, each derived from a separate biological experiment compared to three NSD controls. Although other methods are available to specifically examine methylation of a single candidate gene, no additional validation studies were performed. Examination of the differences in morphology between the tube formation assay results for NSD and HSD EPCs suggested a possible involvement in Notch signaling. The Notch pathway is important in endothelial cells in order to coordinate cellular behaviors during the blood vessel sprouting that occurs in angiogenesis (Hellstrom et al. 2007; Leslie et al. 2007; Siekmann and Lawson 2007). Activation of the Notch pathway is also important for binding of bone marrow progenitor cells to the extracellular matrix (ECM) (Caiado et al. 2008). Caiado et al. (2008) found that the Notch pathway is necessary for adhesion to ECM at sites of vascular repair and that it also regulates the capacity of bone marrow progenitor cells to stimulate angiogenesis and to promote wound healing. They found that bone marrow progenitor cells both express Notch and Notch ligand members. Inhibition of the Notch pathway inhibits bone marrow progenitor cell binding to the extracellular matrix (Caiado et al. 2008). In this study, we injected EPCs into the blood stream as a therapeutic intervention to improve angiogenesis response to electrical stimulation. EPCs injected in this manner are quickly removed from the blood stream. If Notch is important for EPC binding to endothelial cells and basement membrane, it is possible that EPCs homing may depend on Notch signaling. We hypothesized that suppressed Notch in HSD EPCs may account for the inability of HSD EPCs to improve angiogenesis to the same degree as NSD EPCs. We found the suppression of Notch4 in NSD EPCs conferred a phenotype similar to that of HSD EPCs with limited tube formation in vitro. Notch4 suppression in HSD EPCs did not change the phenotype of these cells, showing that Notch4 is important for augmentation of endothelial tube formation by EPCs. Although these studies clearly implicate Notch4 as a candidate in EPC function we did not restore Notch4 levels in HSD EPCs and test the hypothesis that restoration would rescue the angiogenic phenotype. Such an experiment will be critical in the future to determine if elevation of Notch4 could be used in a therapeutic manner.

To the best of our knowledge Notch4 has not been reported as hypermethylated in conditions such as insulin resistance or myocardial infarction. It would be very interesting to determine the methylation status of Notch4 in cardiovascular disease and other common underlying conditions such as hypertension or obesity. Limited studies in adults have demonstrated that Notch4 is expressed in the aorta, endothelial cells of arteries including pulmonary and cardiac vessels and in the endocardium (Iso et al. 2003). Notch4 has also been shown to be suppressed in allografted vessels during the development of transplant arteriosclerosis, as Notch4 is necessary for endothelial quiescence, survival, and repair in response to tissue injury (Quillard et al. 2008). Also Notch4 has been implicated in pregnancy-induced hypertension (Sahin et al. 2011).

Our study also identified other Notch genes including Notch1, Notch2, and Notch3 in our proteomic and DNA methylation analysis, however, none of the other family members met our strict criteria to be a potential candidate. Notch1 protein expression was increased in HSD EPCs than NSD EPCs and had methylated at two distinct spots one in NSD EPCs and the other in HSD EPCs. Notch2 protein expression was also increased in HSD EPCs and had increased methylation in NSD EPCs. Notch3 was only found in NSD EPC protein samples and had three found CpG islands with increased methylation in NSD EPCs. Our proteomics data did not identify delta-like proteins or jagged. This does not mean that these proteins are not expressed in endothelial progenitor cells as they may be at a low expression that was masked during mass spectroscopy. Delta-like and jagged genes were also not identified in the DNA methylation analysis. Further analysis of these genes would be necessary to conclude if there are any differences in expression or methylation between NSD and HSD endothelial progenitor cells.

In summary, high-salt diet altered the DNA methylation profile of EPCs causing a suppressed angiogenic competency. We identified numerous genes with differential methylation and differential protein abundance between NSD and HSD EPCs. Notch4 that emerged as a lead functional candidate had increased methylation in HSD EPCs and suppressed protein expression in HSD EPCs. Notch4 knockdown confirmed its role as necessary in NSD EPC tube formation, revealing one potential mechanism by which high-salt diet suppresses EPC mediated angiogenesis.

## Conflict of Interest

None declared.

## Supporting Information

**Table S1.** Comparison of DNA methylation between NSD and HSD EPCs. **Table S2.** Comparison of proteins found between NSD and HSD EPC samples. **Table S3.** Comparison of gene-protein sets.
