# Supplementary material for: Genome-wide epigenetic and proteomic analysis reveals altered Notch signaling in EPC dysfunction
Source: Physiol Rep. 2015 Apr 28;3(4):e12358. doi: 10.14814/phy2.12358 (PMC4425964; doi:10.14814/phy2.12358)
Supplement: Supplementary file 1 — Table S1. Comparison of DNA methylation between NSD and HSD EPCs. Table S2. Comparison of proteins found between NSD and HSD EPC samples. Table S3. Comparison of gene-protein sets. [file phy20003-e12358-sd1.docx]

**Supplemental Table 1.** Comparison of DNA methylation between NSD and HSD EPCs. The gene symbol, chromosome (Chr), genetic location of the CgG island with start and end sites, NSD methylation rate (NSD methyl), HSD methylation rate (HSD methyl), the log fold change in DNA methylation expression (HSD/NSD), and the p-value between the methylation rates for the NSD and HSD EPCs are listed in the below table. Note: there may be multiple CGI per gene and some CGIs were located in multiple genes. TSS = Transcription start site.

|  |  |  | **CGI Location** | |  |  |  |  |
| --- | --- | --- | --- | --- | --- | --- | --- | --- |
| **Gene Symbol** | **Rat**  **Chr** | **Genetic**  **Location** | **Start** | **End** | **NSD methyl**  **rate** | **HSD methyl**  **rate** | **Log Fold Change** | **P-value** |
| Agap2 | 7 | TSS | 70542496 | 70543655 | 0.5114 | 0.4654 | -0.1844 | 1.62E-12 |
| Ankrd13c | 2 | TSS | 283426054 | 283426974 | 0.0203 | 0.0384 | 0.6572 | 1.39E-35 |
| Areg | 14 | TSS | 18440515 | 18440828 | 0.0036 | 0.0163 | 1.5362 | 1.39E-10 |
| Atmin | 19 | TSS | 60273646 | 60274303 | 0.1975 | 0.2575 | 0.3425 | 4.72E-16 |
| Bai2 | 5 | Intragenic | 151935207 | 151935529 | 0.3910 | 0.4806 | 0.3654 | 1.96E-14 |
| Boll | 9 | TSS | 61783306 | 61784396 | 0.0847 | 0.1168 | 0.3575 | 1.53E-11 |
| Boll | 9 | TSS | 64376479 | 64377569 | 0.0847 | 0.1168 | 0.3575 | 1.53E-11 |
| C1qtnf4 | 3 | Intragenic | 86426075 | 86427064 | 0.1540 | 0.0980 | -0.5162 | 4.04E-33 |
| C4a, Stk19 | 20 | Intragenic | 6524408 | 6525035 | 0.1017 | 0.1451 | 0.4047 | 1.68E-13 |
| C4a, Stk19 | 20 | Intragenic | 6474527 | 6474966 | 0.0697 | 0.1199 | 0.5980 | 6.89E-14 |
| Cd99l2 | 15 | Intragenic | 9795275 | 9795859 | 0.2963 | 0.4577 | 0.6951 | 1.86E-52 |
| Cd99l2 | 15 | Intragenic | 9392929 | 9393513 | 0.2996 | 0.4605 | 0.6905 | 5.05E-49 |
| Chrd | 11 | Intragenic | 86937667 | 86938037 | 0.0193 | 0.0522 | 1.0303 | 9.94E-11 |
| Chrna10 | 1 | Intragenic | 173397510 | 173397895 | 0.7101 | 0.5743 | -0.5961 | 2.03E-12 |
| Ckap2 | 16 | TSS | 74383597 | 74383956 | 0.0824 | 0.1142 | 0.3619 | 4.45E-13 |
| Ckap4 | 7 | TSS | 25084291 | 25085014 | 0.0186 | 0.0049 | -1.3428 | 4.39E-20 |
| Coro1c | 12 | TSS | 50264441 | 50265618 | 0.0035 | 0.0078 | 0.8024 | 1.12E-11 |
| Crb2 | 3 | Intragenic | 27284578 | 27284979 | 0.0666 | 0.0340 | -0.7079 | 1.17E-10 |
| Crip1 | 6 | TSS | 146959722 | 146960185 | 0.0230 | 0.1350 | 1.8911 | 2.49E-15 |
| Cul9, Rps19 | 9 | TSS | 15711796 | 15712572 | 0.1409 | 0.0906 | -0.4981 | 6.52E-26 |
| Cygb | 10 | TSS | 105290264 | 105290535 | 0.0400 | 0.0209 | -0.6688 | 6.74E-11 |
| Ddx54 | 12 | TSS | 43342667 | 43343428 | 0.0008 | 0.0082 | 2.2783 | 1.07E-10 |
| Dip2a | 20 | TSS | 15200815 | 15201648 | 0.0036 | 0.0125 | 1.2562 | 8.51E-18 |
| Dmrta2 | 5 | Intragenic | 133589978 | 133591090 | 0.1773 | 0.2225 | 0.2839 | 3.71E-28 |
| Dusp14 | 10 | TSS | 71296846 | 71297301 | 0.0881 | 0.1291 | 0.4279 | 4.86E-38 |
| Dusp9 | 1 | TSS | 152920567 | 152921701 | 0.0112 | 0.0620 | 1.7632 | 2.03E-14 |
| Ednra | 19 | TSS | 44812449 | 44813149 | 0.0314 | 0.0143 | -0.8077 | 2.3E-19 |
| Etaa1 | 14 | TSS | 102047473 | 102047773 | 0.3520 | 0.4018 | 0.2124 | 3.29E-14 |
| Ewsr1, Rhbdd | 14 | TSS | 86027771 | 86029030 | 0.0100 | 0.0022 | -1.5435 | 1.73E-10 |
| Fbxo41 | 4 | Intragenic | 181843819 | 181844757 | 0.2730 | 0.3510 | 0.3648 | 5.64E-60 |
| Fem1b | 8 | TSS | 67436732 | 67437768 | 0.0069 | 0.0009 | -2.0890 | 6.96E-14 |
| Ftl | 1 | TSS | 102528260 | 102529181 | 0.4792 | 0.5092 | 0.1199 | 7.29E-11 |
| Fzd5 | 9 | TSS | 71118799 | 71121930 | 0.0802 | 0.0961 | 0.1981 | 3.79E-12 |
| Gadd45a | 4 | TSS | 162570071 | 162570730 | 0.0034 | 0.0096 | 1.0367 | 1.94E-10 |
| Gfer, Noxo1 | 10 | TSS | 13878329 | 13879141 | 0.0353 | 0.0519 | 0.4024 | 6.07E-11 |
| Gnas | 3 | TSS | 178470733 | 178472777 | 0.3791 | 0.4213 | 0.1760 | 7.42E-11 |
| Grin2a | 10 | Intragenic | 4527256 | 4527508 | 0.1736 | 0.1129 | -0.5013 | 5.45E-13 |
| Gtpbp6 | 12 | TSS | 54373723 | 54380063 | 0.1764 | 0.1961 | 0.1294 | 1.22E-10 |
| Hdgfrp2 | 9 | TSS | 9978658 | 9979155 | 0.0299 | 0.0041 | -2.0006 | 1.79E-12 |
| Hs6st1 | 9 | TSS | 42273932 | 42274683 | 0.0093 | 0.0040 | -0.8612 | 2.76E-11 |
| Hus1 | 14 | TSS | 89006038 | 89006333 | 0.0078 | 0.0320 | 1.4370 | 4.42E-13 |
| Irgc | 1 | Intragenic | 82554558 | 82555656 | 0.7052 | 0.7735 | 0.3560 | 2.96E-11 |
| Irx4 | 1 | Intragenic | 34065743 | 34066116 | 0.2033 | 0.1259 | -0.5713 | 1.47E-11 |
| Kcna3 | 2 | TSS | 229232471 | 229233779 | 0.0030 | 0.0124 | 1.4237 | 2.15E-11 |
| Kcnb1 | 3 | TSS | 170094018 | 170095983 | 0.1251 | 0.0835 | -0.4508 | 1.45E-20 |
| Kcne1l | X | TSS | 112033994 | 112034575 | 0.3906 | 0.5329 | 0.5764 | 1.45E-12 |
| Khdrbs1 | 5 | TSS | 151679528 | 151681098 | 0.0070 | 0.0027 | -0.9750 | 4.04E-11 |
| Kpna2 | 10 | TSS | 94952577 | 94953144 | 0.0029 | 0.0135 | 1.5413 | 2.25E-13 |
| Krtcap3 | 6 | TSS | 36305513 | 36305894 | 0.6918 | 0.5954 | -0.4220 | 1.59E-21 |
| Lca5l | 11 | Intragenic | 40043628 | 40044228 | 0.0972 | 0.1350 | 0.3714 | 4.92E-13 |
| Llgl2 | 10 | Intragenic | 103887067 | 103887590 | 0.5411 | 0.3705 | -0.6943 | 1.55E-15 |
| LOC310926 | 14 | Intragenic | 46834596 | 46835189 | 0.1627 | 0.1736 | 0.0780 | 4.15E-58 |
| LOC310926 | 14 | Intragenic | 46855715 | 46858996 | 0.2070 | 0.2152 | 0.0496 | 0 |
| LOC310926 | 14 | TSS | 46808853 | 46818337 | 0.1932 | 0.2009 | 0.0485 | 0 |
| Rn45s, Rn5-8s | 14 | TSS | 46822861 | 46824785 | 0.2052 | 0.2172 | 0.0719 | 0 |
| LOC498154 | 12 | Intragenic | 19353137 | 19353439 | 0.0039 | 0.0145 | 1.3278 | 1.35E-11 |
| Ltbp4 | 1 | Intragenic | 85352677 | 85352973 | 0.0635 | 0.0097 | -1.9348 | 5.83E-13 |
| Luc7l2 | 4 | Intragenic | 66148014 | 66148457 | 0.5481 | 0.4458 | -0.4105 | 5.17E-37 |
| Map2k2 | 7 | TSS | 11645565 | 11646037 | 0.0061 | 0.0264 | 1.4790 | 4.54E-13 |
| Map2k5 | 8 | TSS | 68009590 | 68009915 | 0.0122 | 0.0022 | -1.7016 | 1.49E-10 |
| Map4k5, Atl1 | 6 | TSS | 101678102 | 101679036 | 0.0072 | 0.0024 | -1.1030 | 9.82E-13 |
| Med20, Bysl | 9 | TSS | 14314469 | 14315150 | 0.0078 | 0.0010 | -2.0242 | 2.24E-11 |
| Med25 | 1 | TSS | 101952150 | 101952522 | 0.0005 | 0.0095 | 3.0255 | 5.12E-19 |
| Mei1 | 7 | TSS | 123291420 | 123291744 | 0.1147 | 0.2419 | 0.9012 | 9.52E-17 |
| Mest | 4 | TSS | 57810934 | 57811441 | 0.4086 | 0.3103 | -0.4292 | 2.89E-11 |
| Mgat4b | 10 | TSS | 35486971 | 35487295 | 0.0017 | 0.0147 | 2.1630 | 1.55E-14 |
| Minpp1 | 1 | TSS | 258275973 | 258276881 | 0.0057 | 0.0229 | 1.4037 | 8.56E-29 |
| Mir3597-1, Mir9-1 | 2 | TSS | 206984683 | 206985817 | 0.0441 | 0.0260 | -0.5468 | 4.52E-12 |
| Mn1 | 12 | TSS | 52989607 | 52993617 | 0.1245 | 0.1381 | 0.1192 | 9.02E-12 |
| Naa25 | 12 | TSS | 42546997 | 42547634 | 0.1044 | 0.0702 | -0.4342 | 1.23E-17 |
| Neurl1b | 10 | Intragenic | 16947665 | 16948340 | 0.6770 | 0.5897 | -0.3773 | 5.87E-24 |
| Nfatc1 | 18 | Intragenic | 76420811 | 76421924 | 0.0052 | 0.0112 | 0.7818 | 6.41E-15 |
| Nfix | 19 | Intragenic | 36874250 | 36875185 | 0.2208 | 0.1796 | -0.2577 | 1.05E-12 |
| Ngef | 9 | TSS | 94322254 | 94323542 | 0.0122 | 0.0276 | 0.8285 | 9.43E-14 |
| Nmrk1, Ostf1 | 1 | TSS | 241848957 | 241849806 | 0.1052 | 0.0677 | -0.4820 | 2.16E-26 |
| Nnat | 3 | TSS | 161555272 | 161555884 | 0.4535 | 0.3127 | -0.6008 | 4.53E-44 |
| Nr2f2 | 1 | Intragenic | 132490997 | 132491402 | 0.1579 | 0.2466 | 0.5568 | 6.53E-15 |
| Nr4a2 | 3 | TSS | 48187832 | 48188315 | 0.4371 | 0.3891 | -0.1980 | 3.43E-11 |
| Nrg2 | 18 | Intragenic | 28595405 | 28596101 | 0.0405 | 0.0620 | 0.4468 | 7.33E-11 |
| Nyx | X | Intragenic | 10996970 | 10997748 | 0.3855 | 0.5012 | 0.4713 | 9E-12 |
| Oprd1 | 5 | Intragenic | 153962935 | 153963431 | 0.0223 | 0.0089 | -0.9266 | 1.92E-10 |
| Otp | 2 | TSS | 43712119 | 43712321 | 0.1223 | 0.0635 | -0.7203 | 9.74E-11 |
| Pak7 | 3 | TSS | 135840731 | 135840942 | 0.0873 | 0.0208 | -1.5028 | 3.76E-14 |
| Pard6b | 3 | Intragenic | 170987393 | 170988009 | 0.6667 | 0.6133 | -0.2325 | 5.32E-12 |
| Pcdhga1 | 18 | TSS | 30502906 | 30503123 | 0.4573 | 0.3220 | -0.5735 | 9.74E-20 |
| Plekhh3 | 10 | Intragenic | 88876235 | 88876696 | 0.6345 | 0.7263 | 0.4243 | 1.67E-22 |
| Ppp1r12c | 1 | TSS | 75647799 | 75648663 | 0.0026 | 0.0103 | 1.3911 | 1.69E-10 |
| Ppp1r14a | 1 | TSS | 88407248 | 88407770 | 0.0063 | 0.0174 | 1.0198 | 2E-12 |
| Ppp1r2 | 11 | Intragenic | 75957124 | 75957655 | 0.1139 | 0.0780 | -0.4180 | 1.47E-13 |
| Ppp1r3d | 3 | TSS | 183137215 | 183138503 | 0.0156 | 0.0263 | 0.5299 | 8.63E-12 |
| Prep | 20 | TSS | 51788952 | 51789566 | 0.0021 | 0.0081 | 1.3424 | 2.83E-12 |
| Prpf18 | 17 | Intragenic | 79284767 | 79285614 | 0.7159 | 0.7665 | 0.2644 | 4.89E-13 |
| Ptma | 9 | TSS | 93271601 | 93275072 | 0.1331 | 0.1102 | -0.2151 | 6.35E-16 |
| Ptpdc1 | 17 | TSS | 18389064 | 18389824 | 0.0033 | 0.0164 | 1.6029 | 1.11E-15 |
| Ralgapa1 | 6 | Intragenic | 86184745 | 86185062 | 0.5052 | 0.5442 | 0.1564 | 1.17E-11 |
| Rapgef2 | 2 | Intragenic | 197176063 | 197176269 | 0.6423 | 0.7190 | 0.3537 | 4.72E-11 |
| Rere | 5 | TSS | 170960367 | 170961171 | 0.0026 | 0.0092 | 1.2752 | 1.8E-10 |
| Ret | 4 | Intragenic | 216155909 | 216156180 | 0.1691 | 0.3285 | 0.8763 | 6.27E-16 |
| RGD1303003 | 20 | TSS | 13413860 | 13414416 | 0.0028 | 0.0151 | 1.7004 | 3.07E-11 |
| RGD1309821 | 5 | Intragenic | 62404422 | 62406243 | 0.2680 | 0.1915 | -0.4354 | 1.24E-13 |
| RGD1564093 | 19 | TSS | 37118334 | 37118980 | 0.3730 | 0.3233 | -0.2190 | 1.05E-17 |
| Rgl1, Arpc5 | 13 | TSS | 75145329 | 75146573 | 0.0325 | 0.0220 | -0.4007 | 1.04E-11 |
| Rn5-8s | 6 | TSS | 39271041 | 39273061 | 0.1940 | 0.2000 | 0.0383 | 4.9E-137 |
| Rn5-8s | 14 | TSS | 46861592 | 46863445 | 0.1925 | 0.1985 | 0.0384 | 9.76E-64 |
| Rpl13 | 19 | TSS | 66622706 | 66623717 | 0.4046 | 0.3644 | -0.1701 | 2.48E-29 |
| Rps10 | 20 | TSS | 9422355 | 9422826 | 0.0824 | 0.1512 | 0.6842 | 1.47E-40 |
| Rps17 | 1 | TSS | 144111768 | 144112445 | 0.4346 | 0.3852 | -0.2045 | 1.17E-10 |
| Rps19 | 1 | TSS | 83007498 | 83008274 | 0.1330 | 0.0873 | -0.4722 | 1.27E-21 |
| Rrnad1, Isg20l2 | 2 | TSS | 206704944 | 206705426 | 0.2850 | 0.2005 | -0.4632 | 5.56E-41 |
| RT1-CE14, Nfkbil1 | 20 | TSS | 6905202 | 6905445 | 0.0149 | 0.0542 | 1.3326 | 4.67E-14 |
| RT1-CE16, RT1-A3 | 20 | TSS | 5875564 | 5876591 | 0.0140 | 0.0239 | 0.5444 | 1.63E-11 |
| Rtn4rl2 | 3 | Intragenic | 78793261 | 78794062 | 0.0931 | 0.0593 | -0.4874 | 9.43E-22 |
| Scaf11 | 7 | TSS | 137487754 | 137489545 | 0.0758 | 0.0585 | -0.2774 | 6.9E-17 |
| Sept5, Gp1bb | 11 | TSS | 89620816 | 89621926 | 0.2096 | 0.2540 | 0.2496 | 1.33E-14 |
| Shank1 | 1 | Intragenic | 101407980 | 101408357 | 0.1720 | 0.2447 | 0.4441 | 2.06E-16 |
| Shc2 | 7 | TSS | 13117462 | 13118375 | 0.0097 | 0.0031 | -1.1306 | 2.73E-12 |
| Slc10a4 | 14 | TSS | 37580053 | 37580710 | 0.0131 | 0.0242 | 0.6233 | 4.84E-13 |
| Slc12a2 | 18 | TSS | 52103744 | 52105252 | 0.0032 | 0.0073 | 0.8254 | 9.24E-11 |
| Slc25a21 | 6 | TSS | 87636121 | 87636542 | 0.1015 | 0.1371 | 0.3408 | 6.55E-12 |
| Slc8a2 | 1 | Intragenic | 79320156 | 79320410 | 0.1246 | 0.2680 | 0.9440 | 5.42E-18 |
| Smarcc1 | 8 | TSS | 117561242 | 117562175 | 0.0465 | 0.0941 | 0.7550 | 2.89E-39 |
| Snx13 | 6 | TSS | 64111746 | 64112708 | 0.0089 | 0.0026 | -1.2286 | 5.8E-14 |
| Sstr5 | 10 | TSS | 14669507 | 14670255 | 0.0350 | 0.0721 | 0.7624 | 6.5E-11 |
| Stam2 | 3 | TSS | 43377852 | 43378550 | 0.0242 | 0.0553 | 0.8584 | 2.62E-18 |
| Stk17b | 9 | TSS | 60115243 | 60115733 | 0.0166 | 0.0045 | -1.3230 | 8.31E-15 |
| Sult1c2a | 9 | Intragenic | 3229682 | 3230106 | 0.1150 | 0.1297 | 0.1367 | 2.18E-14 |
| Tbx1 | 11 | Intragenic | 89653608 | 89654732 | 0.0996 | 0.0740 | -0.3250 | 3.62E-15 |
| Tbxa2r | 7 | Intragenic | 11421547 | 11422229 | 0.6114 | 0.6622 | 0.2199 | 1.62E-12 |
| Tfap2e | 5 | TSS | 148531334 | 148532636 | 0.0183 | 0.0289 | 0.4661 | 5.49E-14 |
| Tfr2 | 12 | Intragenic | 24194676 | 24194912 | 0.1443 | 0.0486 | -1.1933 | 3.6E-11 |
| Timm23, Parg | 16 | TSS | 8266882 | 8267712 | 0.1153 | 0.1398 | 0.2205 | 1.98E-11 |
| Tmem11 | 10 | TSS | 46904555 | 46905402 | 0.0065 | 0.0016 | -1.4196 | 1.72E-12 |
| Tmem168 | 4 | TSS | 39868087 | 39868575 | 0.0028 | 0.0291 | 2.3579 | 2.31E-15 |
| Tmem59l | 16 | TSS | 20546189 | 20546523 | 0.0241 | 0.0032 | -2.0474 | 1.92E-10 |
| Tnfaip8 | 18 | Intragenic | 43961640 | 43962058 | 0.0344 | 0.0037 | -2.2704 | 4.7E-11 |
| Tspan4 | 1 | Intragenic | 220942880 | 220943379 | 0.0482 | 0.0731 | 0.4426 | 1.41E-16 |
| Tspan4 | 1 | Intragenic | 221379669 | 221380168 | 0.0482 | 0.0731 | 0.4426 | 1.41E-16 |
| Ttc13 | 19 | Intragenic | 68136301 | 68136831 | 0.9286 | 0.8819 | -0.5555 | 7.5E-11 |
| Ttyh3 | 12 | TSS | 18079580 | 18080229 | 0.0084 | 0.0028 | -1.0860 | 1.23E-10 |
| Txndc15 | 17 | Intragenic | 11307791 | 11308662 | 0.0625 | 0.0320 | -0.6996 | 1.57E-17 |
| Uap1 | 13 | TSS | 93123460 | 93124214 | 0.0690 | 0.0299 | -0.8766 | 2.75E-79 |
| Uba6 | 14 | TSS | 23412075 | 23412485 | 0.0011 | 0.0120 | 2.4032 | 2.02E-13 |
| Ublcp1 | 10 | TSS | 29951657 | 29952375 | 0.0108 | 0.0033 | -1.1950 | 1.42E-12 |
| Vps13b | 7 | TSS | 74283545 | 74284233 | 0.0020 | 0.0089 | 1.5058 | 2.98E-16 |
| Vps13b | 7 | Intragenic | 74495436 | 74495825 | 0.3821 | 0.4476 | 0.2702 | 1.27E-11 |
| Wars | 6 | TSS | 141972067 | 141972588 | 0.1620 | 0.1235 | -0.3162 | 1.13E-11 |
| Wdhd1, Socs4 | 15 | TSS | 27996383 | 27997182 | 0.0026 | 0.0080 | 1.1259 | 1.44E-10 |
| Wdyhv1 | 7 | TSS | 98581700 | 98582210 | 0.0102 | 0.0018 | -1.7378 | 4.16E-14 |
| Zc3h13 | 15 | TSS | 61051432 | 61051798 | 0.0018 | 0.0094 | 1.6366 | 4.14E-12 |
| Zfp316 | 12 | Intragenic | 15403031 | 15405129 | 0.2099 | 0.1795 | -0.1940 | 1.2E-16 |

**Supplemental Table 2.** Comparison of proteins found between NSD and HSD EPC samples. The table contains the uniprot ID, description of the protein identified, the number of peptides and scans seen for NSD and HSD samples for each protein, the normalized log ratio between HSD and NSD samples ( = number of HSD scans x ( [Total number scans in NSD x (1/total number scans in HSD) ] / Number of scans in NSD), and the p-value. Proteins only found in NSD EPC samples are labeled as NSD only. Proteins only found in HSD EPC samples are labeled as HSD only.

|  |  | **NSD** | | **HSD** | |  |  |
| --- | --- | --- | --- | --- | --- | --- | --- |
| **Uniprot ID** | **Description** | **Peptide** | **Scans** | **Peptide** | **Scans** | **Normalized log Ratio** | **P-value** |
| P35213 | 14-3-3 protein beta/alpha | 2 | 43 | 0 | 0 | NSD only | 9.36E-19 |
| Q64591 | 2,4-dienoyl-CoA reductase | 5 | 70 | 9 | 212 | 1.0308 | 4.69E-08 |
| P29266 | 3-hydroxyisobutyrate dehydrogenase | 3 | 43 | 0 | 0 | NSD only | 9.36E-19 |
| Q8CJ27 | Abnormal spindle-like microcephaly-associated protein homolog | 7 | 26 | 0 | 0 | NSD only | 6.16E-12 |
| Q8CJ27 | Acyltransferase-like 1-A | 5 | 167 | 4 | 112 | -1.1442 | 4.70E-11 |
| P38983 | ADAMTS-12 precursor | 3 | 27 | 0 | 0 | NSD only | 2.44E-12 |
| Q9WUS0 | Adenylate kinase isoenzyme 2 | 0 | 0 | 2 | 38 | HSD only | 3.86E-10 |
| Q62848 | ADP-ribosylation factor 4 | 0 | 0 | 4 | 193 | HSD only | 3.42E-45 |
| D3ZPP2 | ADP-ribosylation factor-like protein 8A | 2 | 26 | 0 | 0 | NSD only | 6.16E-12 |
| Q80WT5 | Aftiphilin | 3 | 26 | 0 | 0 | NSD only | 6.16E-12 |
| P97275 | Alkyldihydroxyacetonephosphate synthase | 2 | 51 | 0 | 0 | NSD only | 5.97E-22 |
| Q8VHE9 | All-trans-retinol 13,14-reductase | 3 | 8 | 2 | 72 | 2.6021 | 1.32E-09 |
| Q9Z1P2 | Alpha-actinin-1 | 7 | 113 | 17 | 336 | 1.0043 | 1.66E-11 |
| P04764 | Alpha-enolase | 4 | 76 | 2 | 16 | -2.8158 | 6.97E-17 |
| P04764 | Angiogenic factor with G patch and FHA domains 1 | 0 | 0 | 3 | 34 | HSD only | 3.20E-09 |
| P36370 | Antigen peptide transporter 2 | 2 | 25 | 4 | 178 | 2.264 | 1.32E-18 |
| Q63764 | Antizyme inhibitor 2 | 0 | 0 | 3 | 31 | HSD only | 1.57E-08 |
| Q7TMA5 | Apolipoprotein E | 4 | 43 | 0 | 0 | NSD only | 9.36E-19 |
| P00507 | Aspartate aminotransferase | 6 | 54 | 9 | 328 | 2.0348 | 4.74E-29 |
| P19511 | ATP synthase B chain | 5 | 64 | 8 | 370 | 1.9635 | 3.58E-31 |
| P35434 | ATP synthase delta chain | 0 | 0 | 2 | 59 | HSD only | 6.19E-15 |
| P35435 | ATP synthase gamma | 4 | 46 | 13 | 316 | 2.2124 | 6.01E-31 |
| P11608 | ATP synthase protein 8 | 0 | 0 | 2 | 43 | HSD only | 2.76E-11 |
| P19511 | ATP synthase subunit alpha | 15 | 355 | 27 | 1405 | 1.4168 | 1.43E-72 |
| P21571 | ATP synthase-coupling factor 6 | 5 | 320 | 3 | 202 | -1.2316 | 4.19E-22 |
| P41233 | ATP-binding cassette sub-family A member 1 | 4 | 59 | 3 | 25 | -1.8066 | 2.89E-08 |
| P34158 | ATP-binding cassette sub-family D member 3 | 3 | 124 | 3 | 18 | -3.3521 | 1.93E-31 |
| Q9JKB5 | AT-rich interactive domain-containing protein 3A | 4 | 31 | 0 | 0 | NSD only | 6.00E-14 |
| Q5FWU3 | Autophagy-related protein 9A | 3 | 65 | 0 | 0 | NSD only | 1.57E-27 |
| Q05793 | Basement membrane-specific heparan sulfate proteoglycan core protein precursor | 13 | 112 | 29 | 684 | 2.0426 | 5.55E-59 |
| Q6AY58 | B-cell receptor-associated protein 31 | 4 | 31 | 7 | 136 | 1.5654 | 1.65E-09 |
| Q05175 | Brain acid soluble protein1 | 11 | 256 | 5 | 57 | -2.735 | 2.43E-51 |
| Q9QZH2 | BRCA1-associated RING domain protein 1 | 0 | 0 | 8 | 25 | HSD only | 3.83E-07 |
| Q6PAJ1 | Breakpoint cluster region protein | 0 | 0 | 2 | 30 | HSD only | 2.67E-08 |
| Q9Z1Y3 | Cadherin-2 precursor | 2 | 51 | 3 | 22 | -1.7808 | 3.30E-07 |
| P49070 | Calcium signal-modulating cyclophilin ligand | 0 | 0 | 3 | 54 | HSD only | 8.51E-14 |
| P15791 | Calcium/calmodulin-dependent protein kinase type II delta chain | 6 | 81 | 5 | 22 | -2.4483 | 1.94E-15 |
| Q8BH59 | Calcium-binding mitochondrial carrier protein Aralar1 | 7 | 201 | 6 | 141 | -1.0794 | 5.91E-12 |
| Q9QXX4 | Calcium-binding mitochondrial carrier protein Aralar2 | 4 | 197 | 6 | 80 | -1.868 | 3.10E-25 |
| Q62717 | Calcium-dependent secretion activator 1 | 6 | 38 | 3 | 8 | -2.8158 | 3.58E-09 |
| P97756 | Calmodulin | 6 | 365 | 5 | 252 | -1.1023 | 3.88E-21 |
| Q07009 | Calpain-2 catalytic subunit | 5 | 83 | 2 | 14 | -3.1355 | 2.58E-20 |
| P04785 | Calreticulin precursor | 11 | 1073 | 17 | 779 | -1.0298 | 8.57E-53 |
| P12369 | cAMP-dependent protein kinase type II-beta regulatory subunit | 2 | 48 | 0 | 0 | NSD only | 9.41E-21 |
| Q6XQG8 | Carbohydrate sulfotransferase 7 | 3 | 37 | 0 | 0 | NSD only | 2.36E-16 |
| Q8R555 | Cartilage acidic protein 1 | 2 | 35 | 0 | 0 | NSD only | 1.49E-15 |
| D3ZM21 | Catechol-O-methyltransferase domain-containing protein 1 | 2 | 10 | 4 | 69 | 2.2187 | 6.13E-08 |
| Q5U302 | Catenin alpha-1 | 8 | 237 | 6 | 111 | -1.6622 | 1.16E-25 |
| P00787 | Cathepsin B precursor | 0 | 0 | 4 | 108 | HSD only | 4.92E-26 |
| Q6AY20 | Cation-dependent mannose-6-phosphate receptor precursor | 4 | 16 | 4 | 91 | 1.9399 | 1.11E-08 |
| P40241 | CD9 antigen | 0 | 0 | 2 | 141 | HSD only | 1.76E-33 |
| P46892 | Cell division cycle 2-like protein kinase 5 | 8 | 36 | 6 | 9 | -2.5679 | 5.03E-08 |
| A0JPQ9 | Chitinase domain-containing protein 1 | 2 | 43 | 0 | 0 | NSD only | 9.36E-19 |
| Q5BJQ6 | Cleavage stimulation factor subunit 2 | 0 | 0 | 4 | 32 | HSD only | 9.23E-09 |
| P12830 | Cleft lip and palate transmembrane protein 1 homolog | 3 | 117 | 3 | 38 | -2.1903 | 5.39E-19 |
| B0BNA5 | Coactosin-like protein | 2 | 70 | 0 | 0 | NSD only | 1.61E-29 |
| D4ABY2 | Coatomer subunit gamma2 | 0 | 0 | 2 | 34 | HSD only | 3.20E-09 |
| P45592 | Cofilin-1 | 3 | 135 | 4 | 97 | -1.0448 | 4.02E-08 |
| O35638 | Cohesin subunit SA-2 | 0 | 0 | 2 | 33 | HSD only | 5.43E-09 |
| O35206 | Collagen alpha-1(XV) chain precursor | 2 | 30 | 8 | 137 | 1.6233 | 5.31E-10 |
| Q9WUW5 | Collagen alpha-1(XVIII) chain precursor | 0 | 0 | 6 | 34 | HSD only | 3.20E-09 |
| P08649 | Complement C4 precursor | 5 | 32 | 0 | 0 | NSD only | 2.38E-14 |
| Q02874 | Core histone macro-H2A.1 | 3 | 34 | 0 | 0 | NSD only | 3.75E-15 |
| Q91ZN1 | Coronin-1A | 2 | 127 | 2 | 45 | -2.0647 | 4.53E-19 |
| E9PVJ1 | Coronin-1C | 2 | 84 | 4 | 20 | -2.6382 | 2.72E-17 |
| O70244 | Cubilin | 4 | 30 | 0 | 0 | NSD only | 1.51E-13 |
| Q68EJ0 | Cytochrome b5 | 0 | 0 | 4 | 94 | HSD only | 7.18E-23 |
| P00406 | Cytochrome c oxidase subunit 2 | 0 | 0 | 3 | 380 | HSD only | 3.28E-87 |
| P10888 | Cytochrome c oxidase subunit 4 isoform 1 | 6 | 53 | 8 | 242 | 1.6231 | 1.55E-16 |
| Q5M9I5 | Cytochrome c1 heme protein, mitochondrial precursor | 2 | 473 | 7 | 293 | -1.2588 | 5.81E-33 |
| Q64654 | Cytochrome P450 51A1 | 4 | 44 | 0 | 0 | NSD only | 3.73E-19 |
| Q63100 | Cytoplasmic dynein 1 light intermediate chain 1 | 4 | 29 | 0 | 0 | NSD only | 3.82E-13 |
| D3ZH41 | Cytoskeleton-associated protein 4 | 15 | 147 | 23 | 845 | 1.9553 | 1.43E-68 |
| O09175 | Cytosol aminopeptidase | 2 | 33 | 0 | 0 | NSD only | 9.45E-15 |
| P54886 | Delta 1-pyrroline-5-carboxylate synthetase | 8 | 13 | 10 | 145 | 2.9116 | 6.98E-20 |
| P47942 | Dihydropyrimidinase-related protein 2 | 5 | 61 | 0 | 0 | NSD only | 6.16E-26 |
| O88797 | Disabled homolog 2 | 5 | 75 | 3 | 13 | -3.0962 | 2.72E-18 |
| Q62724 | DNA replication licensing factor MCM2 | 4 | 38 | 0 | 0 | NSD only | 9.37E-17 |
| Q9WUL0 | DNA topoisomerase 1 | 2 | 25 | 0 | 0 | NSD only | 1.56E-11 |
| P43138 | DNA-(apurinic or apyrimidinic site) lyase 2 | 0 | 0 | 2 | 40 | HSD only | 1.34E-10 |
| P07153 | Dolichyl-diphosphooligosaccharide--protein glycosyltransferase 67 kDasubunit precursor | 22 | 374 | 32 | 1207 | 1.1225 | 2.81E-44 |
| E5S607 | Dolichyl-diphosphooligosaccharide--protein glycosyltransferase subunit | 0 | 0 | 3 | 116 | HSD only | 7.68E-28 |
| E5S6D8 | Dolichyl-diphosphooligosaccharide--protein glycosyltransferase subunit | 7 | 51 | 14 | 421 | 2.4774 | 3.39E-46 |
| P51400 | Double-stranded RNA-specific editase 1 | 3 | 25 | 0 | 0 | NSD only | 1.56E-11 |
| P38650 | Dynein heavy chain, cytosolic | 26 | 418 | 20 | 296 | -1.0658 | 9.17E-23 |
| Q641Z6 | EH domain-containing protein 1 | 4 | 114 | 0 | 0 | NSD only | 5.34E-47 |
| Q8R3Z7 | EH domain-containing protein 4 | 8 | 173 | 4 | 39 | -2.7171 | 5.18E-35 |
| P13803 | Electron transfer flavoprotein subunit alpha, mitochondrial precursor | 6 | 86 | 12 | 391 | 1.6169 | 1.30E-25 |
| Q569A6 | Endoplasmic reticulum-Golgi intermediate compartment protein 1 | 0 | 0 | 4 | 122 | HSD only | 3.40E-29 |
| P54757 | Ephrin type-A receptor 4 | 0 | 0 | 3 | 30 | HSD only | 2.67E-08 |
| Q569A6 | ER lumen protein retaining receptor 1 | 0 | 0 | 2 | 41 | HSD only | 7.92E-11 |
| B1WBY7 | Erlin-1 precursor | 2 | 35 | 0 | 0 | NSD only | 1.49E-15 |
| Q8R4A1 | ERO1-like protein alpha | 4 | 54 | 4 | 19 | -2.0748 | 5.30E-09 |
| P56571 | ES1 protein homolog, mitochondrial precursor | 2 | 13 | 6 | 84 | 2.124 | 6.02E-09 |
| D3ZPU3 | Estradiol 17-beta-dehydrogenase 12 | 2 | 53 | 9 | 243 | 1.629 | 1.13E-16 |
| Q6P3V8 | Eukaryotic initiation factor 4A-I | 3 | 75 | 0 | 0 | NSD only | 1.65E-31 |
| Q3B8Q2 | Eukaryotic translation initiation factor 3 subunit 4 | 0 | 0 | 4 | 26 | HSD only | 2.24E-07 |
| P31977 | Ezrin | 3 | 41 | 0 | 0 | NSD only | 5.90E-18 |
| P12785 | Fatty acid synthase | 5 | 38 | 0 | 0 | NSD only | 9.37E-17 |
| P30839 | Fatty aldehyde dehydrogenase | 7 | 53 | 6 | 207 | 1.3977 | 7.59E-12 |
| Q62658 | FK506-binding protein 8 | 5 | 206 | 6 | 97 | -1.6544 | 2.25E-22 |
| Q9Z1E1 | Flotillin-1 | 5 | 28 | 6 | 108 | 1.3797 | 9.72E-07 |
| Q9ESQ9 | FMRFamide-related peptides precursor | 3 | 26 | 0 | 0 | NSD only | 6.16E-12 |
| P08050 | Gap junction alpha-1 protein | 4 | 87 | 8 | 59 | -1.1282 | 2.67E-06 |
| P41542 | General vesicular transport factor p115 | 2 | 36 | 0 | 0 | NSD only | 5.93E-16 |
| P50123 | Glutamyl aminopeptidase | 6 | 84 | 12 | 301 | 1.2734 | 1.43E-14 |
| P04041 | Glutathione peroxidase 1 | 4 | 62 | 2 | 31 | -1.5679 | 2.90E-07 |
| Q9ESV6 | Glyceraldehyde-3-phosphate dehydrogenase | 6 | 65 | 10 | 268 | 1.4759 | 5.08E-16 |
| Q9R080 | G-protein-signaling modulator 2 | 0 | 0 | 2 | 26 | HSD only | 2.24E-07 |
| P10824 | Guanine nucleotide-binding protein G(q) subunit alpha | 0 | 0 | 7 | 138 | HSD only | 8.35E-33 |
| Q5XHZ0 | Heat shock protein 75 kDa | 6 | 86 | 5 | 26 | -2.2937 | 3.77E-15 |
| P06762 | Heme oxygenase 1 | 0 | 0 | 5 | 85 | HSD only | 7.81E-21 |
| P23711 | Heme oxygenase 2 | 3 | 41 | 0 | 0 | NSD only | 5.90E-18 |
| P02088 | Hemoglobin subunit beta-1 | 0 | 0 | 4 | 77 | HSD only | 5.07E-19 |
| P27926 | Hexokinase-3 | 3 | 48 | 0 | 0 | NSD only | 9.41E-21 |
| Q63692 | Hsp90 co-chaperone Cdc37 | 3 | 78 | 0 | 0 | NSD only | 1.06E-32 |
| Q63617 | Hypoxia up-regulated protein 1 | 10 | 236 | 14 | 171 | -1.0326 | 6.56E-13 |
| Q91YE6 | Importin-9 | 3 | 48 | 2 | 12 | -2.5679 | 3.10E-10 |
| P29994 | Inositol 1,4,5-trisphosphate receptor type 1 | 0 | 0 | 5 | 47 | HSD only | 3.36E-12 |
| D3ZQM3 | Integrin alpha-3 precursor | 0 | 0 | 2 | 89 | HSD only | 9.71E-22 |
| Q4V8C7 | Interferon-inducible protein | 2 | 42 | 2 | 158 | 1.3436 | 6.38E-09 |
| Q9WVE9 | Intersectin-1 | 2 | 32 | 0 | 0 | NSD only | 2.38E-14 |
| P12007 | Isovaleryl-CoA dehydrogenase | 3 | 77 | 0 | 0 | NSD only | 2.64E-32 |
| Q61595 | Kinectin | 8 | 83 | 13 | 264 | 1.1015 | 1.27E-10 |
| Q2P9S1 | Kinesin-like protein KIF1A | 2 | 12 | 3 | 77 | 2.114 | 2.82E-08 |
| P57016 | Ladinin-1 | 4 | 30 | 0 | 0 | NSD only | 1.51E-13 |
| P11048 | Lamin-A | 4 | 68 | 20 | 205 | 1.0242 | 9.10E-08 |
| Q61029 | Lamina-associated polypeptide 2 isoform beta | 2 | 40 | 0 | 0 | NSD only | 1.48E-17 |
| Q80U72 | LAP4 protein | 0 | 0 | 5 | 26 | HSD only | 2.24E-07 |
| Q6LDZ3 | Leukocyte common antigen precursor | 10 | 116 | 7 | 59 | -1.5432 | 4.09E-12 |
| D2N7K7 | Lipoamide acyltransferase component of branched-chain alpha-keto aciddehydrogenase complex | 0 | 0 | 4 | 49 | HSD only | 1.18E-12 |
| P18163 | Long-chain fatty acid transport protein 1 | 3 | 81 | 0 | 0 | NSD only | 6.78E-34 |
| P33124 | Long-chain-fatty-acid--CoA ligase 1 | 14 | 171 | 26 | 720 | 1.5061 | 1.77E-41 |
| O88204 | Low-density lipoprotein receptor-related protein 1 | 23 | 295 | 50 | 914 | 1.0636 | 2.77E-31 |
| P17046 | Lysosome-associated membrane glycoprotein 2 | 0 | 0 | 3 | 74 | HSD only | 2.43E-18 |
| P30204 | Macrophage scavenger receptor types I and II | 0 | 0 | 4 | 28 | HSD only | 7.73E-08 |
| Q6AYC4 | Macrophage-capping protein | 5 | 138 | 4 | 65 | -1.654 | 1.69E-15 |
| Q62667 | Major vault protein | 6 | 134 | 4 | 72 | -1.464 | 7.99E-13 |
| O88989 | Malate dehydrogenase | 13 | 258 | 20 | 1061 | 1.4721 | 2.32E-58 |
| P70580 | Membrane-associated progesterone receptor component 1 | 6 | 276 | 7 | 133 | -1.6211 | 1.33E-28 |
| Q5XIT9 | Methylcrotonoyl-CoA carboxylase beta chain | 3 | 43 | 4 | 16 | -1.9941 | 3.94E-07 |
| O35093 | Mitochondrial import inner membrane translocase subunit Tim23 | 4 | 145 | 3 | 83 | -1.3727 | 1.35E-12 |
| Q75Q40 | Mitochondrial import receptor subunit TOM40 homolog | 2 | 29 | 0 | 0 | NSD only | 3.82E-13 |
| Q7TSA0 | Mitochondrial Rho GTPase 1 | 0 | 0 | 4 | 41 | HSD only | 7.92E-11 |
| O88994 | MOSC domain-containing protein 2 | 3 | 71 | 0 | 0 | NSD only | 6.43E-30 |
| Q8CG09 | Multidrug resistance-associated protein 1 | 5 | 89 | 3 | 24 | -2.4586 | 6.84E-17 |
| Q64122 | Myosin light polypeptide 3 | 0 | 0 | 2 | 31 | HSD only | 1.57E-08 |
| Q63356 | Myosin-Ie | 4 | 49 | 5 | 20 | -1.8606 | 2.44E-07 |
| Q561S0 | NADH dehydrogenase [ubiquinone] 1 alpha subcomplex subunit 2 | 0 | 0 | 2 | 26 | HSD only | 2.24E-07 |
| Q5PQZ9 | NADH dehydrogenase [ubiquinone] 1 subunit C2 | 0 | 0 | 3 | 32 | HSD only | 9.23E-09 |
| P19234 | NADH dehydrogenase [ubiquinone] flavoprotein2 | 4 | 109 | 3 | 72 | -1.1661 | 6.71E-08 |
| P20070 | NADH-cytochrome b5 reductase | 7 | 113 | 7 | 57 | -1.5551 | 5.85E-12 |
| Q68EJ0 | NADH-cytochrome b5 reductase 3 | 10 | 201 | 17 | 882 | 1.5657 | 3.24E-53 |
| Q64289 | Neurogenic differentiation factor 4 | 2 | 27 | 0 | 0 | NSD only | 2.44E-12 |
| Q64319 | Neutral amino acid transporter B(0) | 2 | 30 | 0 | 0 | NSD only | 1.51E-13 |
| Q5XIA1 | Nicalin precursor | 0 | 0 | 5 | 38 | HSD only | 3.86E-10 |
| P62961 | Nuclease sensitive element-binding protein 1 | 2 | 48 | 0 | 0 | NSD only | 9.41E-21 |
| Q63083 | Nucleobindin-1 precursor | 5 | 36 | 9 | 174 | 1.7052 | 4.38E-13 |
| P13383 | Nucleolin | 0 | 0 | 3 | 32 | HSD only | 9.23E-09 |
| P24368 | Peptidyl-prolyl cis-trans isomerase B precursor | 7 | 125 | 12 | 454 | 1.2929 | 1.19E-21 |
| P43884 | Perilipin | 0 | 0 | 5 | 60 | HSD only | 3.67E-15 |
| P07896 | Peroxisomal 3,2-trans-enoyl-CoA isomerase | 0 | 0 | 2 | 46 | HSD only | 5.69E-12 |
| P97852 | Peroxisomal multifunctional enzyme type 2 | 5 | 35 | 14 | 152 | 1.5508 | 2.44E-10 |
| Q9JLI4 | Peroxisome proliferator-activated receptor-binding protein | 4 | 33 | 0 | 0 | NSD only | 9.45E-15 |
| P62025 | Phosphatase and actin regulator 2 | 4 | 27 | 0 | 0 | NSD only | 2.44E-12 |
| P16036 | Phosphate carrier protein | 3 | 24 | 15 | 1305 | 5.197 | 0 |
| O70173 | Phosphatidylinositol-4-phosphate 3-kinase C2 domain-containing gammapolypeptide | 0 | 0 | 5 | 30 | HSD only | 2.67E-08 |
| P07379 | Phosphoenolpyruvate carboxykinase [GTP] | 3 | 125 | 6 | 90 | -1.0418 | 1.36E-07 |
| D4A6X7 | Phosphopantothenate--cysteine ligase | 2 | 38 | 0 | 0 | NSD only | 9.37E-17 |
| Q9QXZ7 | Photoreceptor-specific nuclear receptor | 2 | 35 | 0 | 0 | NSD only | 1.49E-15 |
| Q6AYS4 | Plasma alpha-L-fucosidase precursor | 0 | 0 | 2 | 36 | HSD only | 1.11E-09 |
| Q61233 | Plastin-2 | 9 | 184 | 4 | 85 | -1.682 | 1.31E-20 |
| Q9QYM2 | Poly(ADP-ribose) glycohydrolase | 2 | 26 | 0 | 0 | NSD only | 6.16E-12 |
| Q9SX55 | Probable mitochondrial import receptor subunit TOM40 homolog | 0 | 0 | 5 | 53 | HSD only | 1.44E-13 |
| Q8N2H3 | Probable oxidoreductase C10orf33 homolog | 0 | 0 | 2 | 28 | HSD only | 7.73E-08 |
| P62963 | Profilin-1 | 0 | 0 | 4 | 94 | HSD only | 7.18E-23 |
| Q9QZA2 | Programmed cell death 6-interacting protein | 5 | 152 | 0 | 0 | NSD only | 4.59E-62 |
| P67779 | Prohibitin | 9 | 173 | 19 | 741 | 1.5308 | 1.30E-43 |
| Q5XIH7 | Prohibitin-2 | 3 | 68 | 11 | 718 | 2.8325 | 0 |
| P0C5H9 | Protein ARMET precursor | 5 | 43 | 0 | 0 | NSD only | 9.36E-19 |
| Q8BXZ1 | Protein disulfide-isomerase TXNDC10 | 0 | 0 | 7 | 86 | HSD only | 4.64E-21 |
| Q9Z1W6 | Protein LYRIC | 6 | 102 | 5 | 74 | -1.0308 | 2.37E-06 |
| P61621 | Protein transport protein Sec61 subunit alpha isoform 1 | 3 | 108 | 8 | 468 | 1.5476 | 1.36E-28 |
| P57097 | Proto-oncogene tyrosine-protein kinase MER | 0 | 0 | 3 | 29 | HSD only | 4.54E-08 |
| G3V9H8 | Proto-oncogene tyrosine-protein kinase receptor Ret | 5 | 28 | 0 | 0 | NSD only | 9.65E-13 |
| Q9JJ25 | Pyrin | 0 | 0 | 3 | 33 | HSD only | 5.43E-09 |
| Q5PQJ6 | Pyrroline-5-carboxylate reductase 2 | 0 | 0 | 3 | 37 | HSD only | 6.54E-10 |
| Q06437 | Pyruvate dehydrogenase E1 component alpha subunit | 4 | 62 | 9 | 206 | 1.1645 | 2.91E-09 |
| P49432 | Pyruvate dehydrogenase E1 component subunit beta | 2 | 22 | 9 | 158 | 2.2765 | 8.67E-17 |
| Q62921 | RanBP-type and C3HC4-type zinc finger-containing protein 1 | 0 | 0 | 3 | 27 | HSD only | 1.32E-07 |
| Q9JKF1 | Ras GTPase-activating-like protein IQGAP1 | 21 | 583 | 17 | 344 | -1.3289 | 1.13E-43 |
| P35281 | Ras-related protein Rab-10 | 0 | 0 | 3 | 26 | HSD only | 2.24E-07 |
| P61107 | Ras-related protein Rab-14 | 5 | 199 | 7 | 136 | -1.117 | 1.88E-12 |
| Q5EB77 | Ras-related protein Rab-18 | 2 | 49 | 7 | 159 | 1.1303 | 3.50E-07 |
| Q6GQP4 | Ras-related protein Rab-31 | 0 | 0 | 2 | 47 | HSD only | 3.36E-12 |
| Q5U316 | Ras-related protein Rab-35 | 0 | 0 | 2 | 40 | HSD only | 1.34E-10 |
| P61021 | Ras-related protein Rab-5B | 0 | 0 | 3 | 51 | HSD only | 4.11E-13 |
| Q9WVB1 | Ras-related protein Rab-6A | 0 | 0 | 5 | 40 | HSD only | 1.34E-10 |
| P70550 | Ras-related protein Rab-8B | 0 | 0 | 2 | 30 | HSD only | 2.67E-08 |
| Q62636 | Ras-related protein Rap-1b precursor | 8 | 204 | 4 | 113 | -1.4201 | 6.03E-18 |
| Q9JIR4 | Regulating synaptic membrane exocytosis protein 1 | 0 | 0 | 3 | 42 | HSD only | 4.67E-11 |
| Q9JIS1 | Regulating synaptic membrane exocytosis protein 2 | 3 | 29 | 0 | 0 | NSD only | 3.82E-13 |
| Q62703 | Reticulocalbin-2 precursor | 6 | 145 | 6 | 48 | -2.1628 | 8.27E-23 |
| Q9JK11 | Reticulon-4 | 12 | 285 | 10 | 200 | -1.0788 | 2.59E-16 |
| Q5XXR3 | Rho guanine nucleotide exchange factor 6 | 2 | 30 | 0 | 0 | NSD only | 1.51E-13 |
| P62747 | Rho-related GTP-binding protein RhoB precursor | 2 | 43 | 0 | 0 | NSD only | 9.36E-19 |
| Q9Z2J9 | Runt-related transcription factor 2 | 3 | 41 | 0 | 0 | NSD only | 5.90E-18 |
| Q62991 | Sec1 family domain-containing protein 1 | 3 | 33 | 0 | 0 | NSD only | 9.45E-15 |
| P56603 | Secretory carrier-associated membrane protein 1 | 3 | 98 | 2 | 58 | -1.3246 | 1.45E-08 |
| Q9WVC0 | Septin-7 | 4 | 49 | 0 | 0 | NSD only | 3.75E-21 |
| Q9EP89 | Serine beta-lactamase-like protein LACTB | 7 | 55 | 0 | 0 | NSD only | 1.52E-23 |
| O04951 | Serine/threonine-protein phosphatase 2A 65 kDa regulatory subunit Aalpha | 5 | 45 | 3 | 18 | -1.8898 | 5.71E-07 |
| P55159 | Serum paraoxonase/arylesterase 2 | 5 | 37 | 12 | 260 | 2.2451 | 3.45E-26 |
| P42667 | Signal peptidase complex catalytic subunit SEC11A | 0 | 0 | 4 | 67 | HSD only | 9.41E-17 |
| O54861 | Sortilin precursor | 5 | 87 | 2 | 24 | -2.4258 | 2.65E-16 |
| P16975 | SPARC precursor | 0 | 0 | 5 | 42 | HSD only | 4.67E-11 |
| Q5PPL3 | Sterol-4-alpha-carboxylate 3-dehydrogenase, decarboxylating | 0 | 0 | 4 | 61 | HSD only | 2.17E-15 |
| B2GV06 | Succinyl-CoA:3-ketoacid-coenzyme A transferase 1, mitochondrialprecursor | 10 | 94 | 12 | 440 | 1.6589 | 1.32E-29 |
| P07895 | Superoxide dismutase[Mn] | 5 | 52 | 6 | 242 | 1.6506 | 6.80E-17 |
| Q4QQU6 | Survival motor neuron protein | 0 | 0 | 3 | 26 | HSD only | 2.24E-07 |
| Q3MIE4 | Synaptic vesicle membrane protein VAT-1 homolog | 3 | 119 | 6 | 77 | -1.1959 | 8.56E-09 |
| G3V7P1 | Syntaxin-12 | 6 | 86 | 3 | 37 | -1.7847 | 3.13E-11 |
| Q498D4 | Talin-1 | 34 | 992 | 25 | 388 | -1.9221 | 0 |
| D3ZE09 | Talin-2 | 7 | 53 | 8 | 16 | -2.2958 | 6.58E-10 |
| Q99JC6 | Tapasin precursor | 0 | 0 | 2 | 36 | HSD only | 1.11E-09 |
| P05540 | T-cell surface glycoprotein CD4 precursor | 4 | 104 | 0 | 0 | NSD only | 4.97E-43 |
| Q68FQ0 | T-complex protein 1 subunit epsilon | 0 | 0 | 2 | 55 | HSD only | 5.04E-14 |
| Q6P502 | T-complex protein 1 subunit gamma | 3 | 31 | 0 | 0 | NSD only | 6.00E-14 |
| Q5XHX6 | Thioredoxin domain-containing protein 1 | 0 | 0 | 2 | 45 | HSD only | 9.63E-12 |
| Q5XHX6 | Thioredoxin domain-containing protein 4 | 0 | 0 | 4 | 72 | HSD only | 6.90E-18 |
| P49430 | Thromboxane-A synthase | 3 | 28 | 0 | 0 | NSD only | 9.65E-13 |
| Q80YV3 | Transformation/transcription domain-associated protein | 0 | 0 | 5 | 25 | HSD only | 3.83E-07 |
| P61589 | Transforming protein RhoA precursor | 3 | 34 | 5 | 119 | 1.2395 | 2.16E-06 |
| O35433 | Transient receptor potential cation channel subfamily V member 2 | 6 | 122 | 7 | 82 | -1.141 | 2.01E-08 |
| P46462 | Transitional endoplasmic reticulum ATPase | 6 | 80 | 12 | 306 | 1.3676 | 2.63E-16 |
| Q8BU14 | Translocation protein SEC62 | 0 | 0 | 4 | 42 | HSD only | 4.67E-11 |
| Q8VHE0 | Translocation protein SEC63 homolog | 8 | 110 | 4 | 30 | -2.4423 | 2.35E-20 |
| Q07984 | Translocon-associated protein subunit delta precursor | 0 | 0 | 5 | 169 | HSD only | 8.65E-40 |
| Q9QYJ4 | Transmembrane 9 superfamily protein member 1 precursor | 0 | 0 | 2 | 28 | HSD only | 7.73E-08 |
| Q63584 | Transmembrane emp24 domain-containing protein 10 precursor | 3 | 91 | 8 | 334 | 1.3081 | 1.32E-16 |
| Q4V899 | Transmembrane protein 165 | 2 | 42 | 0 | 0 | NSD only | 2.35E-18 |
| Q5XIL0 | Transmembrane protein 167 precursor | 0 | 0 | 3 | 54 | HSD only | 8.51E-14 |
| P32089 | Tricarboxylate transport protein, mitochondrial precursor | 4 | 72 | 8 | 261 | 1.2901 | 4.63E-13 |
| P09495 | Tropomyosin alpha-4 chain | 4 | 46 | 0 | 0 | NSD only | 5.92E-20 |
| P04692 | Tropomyosin beta chain | 0 | 0 | 3 | 72 | HSD only | 6.90E-18 |
| Q5XIF6 | Tubulin alpha-4A chain | 0 | 0 | 2 | 113 | HSD only | 3.65E-27 |
| A2AQ07 | Tubulin beta-1 chain | 11 | 215 | 7 | 36 | -3.1461 | 3.88E-50 |
| Q9EQT5 | Tubulointerstitial nephritis antigen-like precursor | 2 | 71 | 4 | 40 | -1.3957 | 5.04E-07 |
| Q6PCT3 | Tumor protein D54 | 3 | 37 | 0 | 0 | NSD only | 2.36E-16 |
| D3ZDK2 | Ubiquitin-conjugating enzyme E2 O | 0 | 0 | 2 | 54 | HSD only | 8.51E-14 |
| O70199 | UDP-glucose 6-dehydrogenase | 3 | 25 | 0 | 0 | NSD only | 1.56E-11 |
| D3ZDJ4 | UNC93 homolog B1 | 0 | 0 | 2 | 32 | HSD only | 9.23E-09 |
| H0V4E2 | Uncharacterized protein C1orf85 homolog precursor | 0 | 0 | 2 | 35 | HSD only | 1.88E-09 |
| H0V7S3 | Uncharacterized protein KIAA1033 | 0 | 0 | 2 | 28 | HSD only | 7.73E-08 |
| Q80SY3 | Vacuolar ATP synthase subunit D | 3 | 54 | 0 | 0 | NSD only | 3.80E-23 |
| O08700 | Vacuolar protein sorting-associated protein 45 | 2 | 28 | 0 | 0 | NSD only | 9.65E-13 |
| P31000 | Vimentin | 29 | 1106 | 38 | 3451 | 1.0738 | 0 |
| P62762 | Visinin-like protein 1 | 2 | 34 | 0 | 0 | NSD only | 3.75E-15 |
| Q9R1Z0 | Voltage-dependent anion-selective channel protein 3 | 7 | 127 | 0 | 0 | NSD only | 3.73E-52 |
| Q9JMJ4 | WD repeat protein 19 | 2 | 31 | 0 | 0 | NSD only | 6.00E-14 |
| Q6X782 | Zona pellucida-binding protein 2 precursor | 2 | 26 | 0 | 0 | NSD only | 6.16E-12 |

**Supplemental Table 3.** Comparison of gene-protein sets. The table contains gene-protein sets with information on the accession number, gene identification, general location of CpG island, the DNA methylation fold change (HSD/NSD), DNA methylation p-value, protein fold change (HSD/NSD), and the p-value for the protein expression. Proteins only found in NSD EPC samples are labeled as NSD only. Proteins only found in HSD EPC samples are labeled as HSD only. TSS = transcription start site.

| **Accession**  **Number** | **Gene ID** | **Genetic**  **Location** | **DNA Met.**  **Ratio**  **(HSD/NSD)** | **DNA Met.**  **P-value** | **Protein Ratio**  **(HSD/NSD)** | **Protein**  **P-value** |
| --- | --- | --- | --- | --- | --- | --- |
| **P31695** | **Notch4** | **Intragenic** | **2.3984** | **0.0054113** | **NSD only** | **0.002435** |
| **P97953** | **Vegfc** | **TSS** | **1.6053** | **0.0050728** | **NSD only** | **0.002435** |
| **P35546** | **Ret** | **Intragenic** | **2.4021** | **6.27E-16** | **NSD only** | **7.31E-13** |
| P29411 | Ak3 | TSS | 0.2725 | 0.00000633 | 4.2000 | 3.04E-08 |
| O35451 | Atf6b | TSS | 0.4109 | 0.00154794 | HSD only | 5.51E-08 |
| P54258 | Atn1 | Intragenic | 1.8599 | 0.00084921 | NSD only | 3.93E-07 |
| Q64446 | Atp7b | TSS | 2.3095 | 0.00176931 | NSD only | 0.0024346 |
| Q00993 | Axl | Intragenic | 1.8363 | 0.00149777 | NSD only | 0.0024346 |
| Q02435 | Bfsp1 | TSS | 0.6993 | 0.00000384 | HSD only | 6.40E-06 |
| Q9JLK7 | Cabp1 | Intragenic | 1.4899 | 9.08E-08 | NSD only | 0.00033483 |
| Q6AYK6 | Cacybp | TSS | 4.0390 | 0.0000368 | NSD only | 2.28E-08 |
| Q9QZA6 | Cd151 | TSS | 0.3534 | 0.0000769 | 5.5000 | 1.64E-05 |
| Q63686 | Cdk16 | TSS | 2.7229 | 0.00431926 | NSD only | 1.22E-11 |
| Q03059 | Chat | TSS | 1.4033 | 0.00000167 | NSD only | 0.0024346 |
| Q03059 | Chat | Intragenic | 1.2522 | 0.00141982 | NSD only | 0.0024346 |
| Q9CRB9 | Chchd3 | TSS | 0.4385 | 0.00048058 | 5.3125 | 1.44E-07 |
| Q8BMK4 | Ckap4 | TSS | 0.2611 | 4.39E-20 | 5.7483 | 8.44E-67 |
| P51799 | Clcn7 | TSS | 3.0715 | 0.0000852 | NSD only | 0.00012587 |
| Q9Z0W7 | Clic4 | TSS | 2.5439 | 0.0000206 | NSD only | 5.87E-08 |
| P53996 | Cnbp | TSS | 2.6091 | 0.0000692 | NSD only | 0.00089798 |
| Q8BWQ5 | Dclk3 | TSS | 1.6176 | 0.00263628 | NSD only | 1.02E-06 |
| Q91YD3 | Dcp1a | TSS | 2.5832 | 0.0000464 | NSD only | 0.0024346 |
| Q9CWS0 | Ddah1 | TSS | 1.5337 | 0.00430681 | NSD only | 1.22E-11 |
| Q64591 | Decr1 | TSS | 0.4209 | 0.00264358 | 3.0286 | 9.64E-08 |
| Q9Z2A7 | Dgat1 | TSS | 3.1239 | 0.0001864 | NSD only | 1.02E-06 |
| Q6PFD5 | Dlgap3 | Intragenic | 2.2573 | 0.0005183 | NSD only | 0.00033483 |
| Q8C147 | Dock8 | TSS | 1.9184 | 0.00014239 | NSD only | 4.76E-05 |
| Q8BLD9 | Drd5 | TSS | 1.3875 | 0.0000707 | NSD only | 0.00089798 |
| Q64623 | Dusp1 | TSS | 2.1328 | 0.00425991 | NSD only | 0.00089798 |
| Q9WVK4 | Ehd1 | TSS | 2.1135 | 0.00023165 | NSD only | 1.76E-47 |
| Q6PDJ6 | Fbxo42 | TSS | 1.6753 | 0.00408686 | NSD only | 0.00089798 |
| Q69ZL1 | Fgd6 | TSS | 1.7892 | 0.00523016 | NSD only | 7.95E-11 |
| Q91V87 | Fgfrl1 | TSS | 0.5495 | 0.0000512 | HSD only | 9.31E-05 |
| Q9JHE4 | Gal3st1 | TSS | 0.7556 | 0.00000124 | HSD only | 0.00001091 |
| P56213 | Gfer | TSS | 1.4955 | 6.07E-11 | NSD only | 6.92E-06 |
| Q8R4A8 | Gnas | TSS | 1.1925 | 7.42E-11 | NSD only | 1.81E-05 |
| Q02874 | H2afy | TSS | 2.0722 | 1.92E-07 | NSD only | 2.68E-15 |
| Q8BUK6 | Hook3 | TSS | 1.8730 | 0.0000616 | NSD only | 0.00033483 |
| Q6P7R8 | Hsd17b12 | TSS | 0.0735 | 0.00077964 | 4.5849 | 3.32E-16 |
| P11499 | Hsp90ab1 | TSS | 1.9994 | 0.00105127 | NSD only | 1.74E-14 |
| P14659 | Hspa2 | TSS | 1.4038 | 1.89E-07 | NSD only | 7.31E-13 |
| Q9D6R2 | Idh3a | TSS | 2.1220 | 0.0000423 | NSD only | 7.31E-13 |
| Q63269 | Itpr3 | Intragenic | 0.5717 | 1.65E-08 | HSD only | 5.43E-05 |
| Q9QXX0 | Jag1 | Intragenic | 0.3692 | 0.00035781 | HSD only | 0.00081805 |
| Q62137 | Jak3 | Intragenic | 2.4173 | 0.00018095 | NSD only | 0.00089798 |
| O88447 | Klc1 | TSS | 1.9588 | 0.0025227 | NSD only | 0.00033483 |
| Q6P6Q2 | Krt5 | TSS | 0.8264 | 0.000081 | 17.6667 | 1.99E-17 |
| Q9EP89 | Lactb | TSS | 2.1565 | 0.00507341 | NSD only | 8.83E-24 |
| P48538 | Lgals1 | Intragenic | 0.0000 | 0.00231366 | HSD only | 0.0014185 |
| Q91VR7 | Map1lc3a | TSS | 1.6524 | 0.00258139 | NSD only | 0.00033483 |
| P60756 | Mdga2 | TSS | 1.4269 | 4.81E-07 | NSD only | 0.00012587 |
| Q719N3 | Mmd | TSS | 1.8513 | 0.00020825 | NSD only | 2.65E-06 |
| P33434 | Mmp2 | TSS | 1.2364 | 0.00286887 | NSD only | 1.81E-05 |
| Q8VI63 | Mob2 | TSS | 0.5770 | 0.00036735 | HSD only | 6.40E-06 |
| Q5I0C5 | Mtfmt | TSS | 1.8990 | 0.00285042 | NSD only | 0.00089798 |
| Q9WTI7 | Myo1c | TSS | 0.5930 | 0.00104618 | 2.2379 | 3.41E-08 |
| Q8BLF1 | Nceh1 | TSS | 2.6373 | 0.00302114 | NSD only | 0.00089798 |
| Q9CR21 | Ndufab1 | TSS | 0.7478 | 0.00086195 | HSD only | 2.67E-07 |
| Q9CQ54 | Ndufc2 | TSS | 0.1595 | 1.53E-07 | HSD only | 1.14E-08 |
| O88942 | Nfatc1 | Intragenic | 2.1854 | 6.41E-15 | NSD only | 0.00089798 |
| P54729 | Nub1 | TSS | 3.0803 | 0.00000421 | NSD only | 2.65E-06 |
| Q9QYM2 | Parg | TSS | 1.2466 | 1.98E-11 | NSD only | 4.76E-12 |
| Q8CFI5 | Pars2 | TSS | 0.4925 | 0.00196622 | HSD only | 9.31E-05 |
| P52873 | Pc | TSS | 0.3306 | 0.00123609 | 2.4177 | 2.63E-07 |
| O54735 | Pde5a | TSS | 1.4971 | 0.00043432 | NSD only | 0.00012587 |
| Q5XIH7 | Phb2 | TSS | 0.3939 | 0.00111528 | 10.5588 | 1.65E-87 |
| Q5SPL2 | Phf12 | TSS | 1.9292 | 0.0000938 | NSD only | 3.44E-09 |
| Q8R2H9 | Phospho1 | Intragenic | 0.7993 | 0.00393259 | HSD only | 0.0002746 |
| P30427 | Plec | TSS | 0.4872 | 0.0012027 | 2.0716 | 1.27E-12 |
| Q9CQ36 | Pole4 | TSS | 3.0555 | 0.0000205 | NSD only | 0.00033483 |
| P25321 | Prkaca | TSS | 1.8382 | 0.00044978 | NSD only | 1.34E-09 |
| O08762 | Prss12 | TSS | 1.5620 | 0.0000951 | NSD only | 0.00012587 |
| Q63347 | Psmc2 | TSS | 7.6168 | 0.0000533 | NSD only | 2.65E-06 |
| Q53B90 | Rab43 | TSS | 1.8057 | 0.00230068 | NSD only | 7.31E-13 |
| Q99MK9 | Rassf1 | TSS | 0.4786 | 0.00271971 | HSD only | 0.00015969 |
| Q9JLT7 | Rax | TSS | 1.3692 | 0.0000372 | NSD only | 0.00089798 |
| Q60695 | Rgl1 | TSS | 0.6699 | 1.04E-11 | HSD only | 3.18E-05 |
| Q80U40 | Rimbp2 | Intragenic | 1.2773 | 0.0011515 | NSD only | 0.00089798 |
| Q9EQZ7 | Rims2 | TSS | 1.2599 | 0.00136543 | NSD only | 2.87E-13 |
| Q9D684 | Rin2 | Intragenic | 2.5229 | 0.00079837 | NSD only | 0.00089798 |
| Q63644 | Rock1 | TSS | 2.0528 | 0.0000227 | NSD only | 3.44E-09 |
| P41123 | Rpl13 | TSS | 0.8436 | 2.48E-29 | 7.9231 | 5.87E-12 |
| P12001 | Rpl18 | TSS | 0.3313 | 0.00443457 | 6.7857 | 4.53E-10 |
| P84100 | Rpl19 | TSS | 0.1477 | 0.00418109 | 8.4286 | 1.04E-07 |
| P61354 | Rpl27 | TSS | 0.7552 | 5.55E-07 | HSD only | 0.0002746 |
| P63325 | Rps10 | TSS | 1.9823 | 1.47E-40 | NSD only | 3.44E-09 |
| P17074 | Rps19 | TSS | 0.6250 | 1.27E-21 | 3.2600 | 3.98E-07 |
| P60868 | Rps20 | TSS | 0.9029 | 0.00018105 | HSD only | 1.07E-19 |
| P62852 | Rps25 | TSS | 3.4814 | 0.00276377 | NSD only | 0.00033483 |
| P62242 | Rps8 | TSS | 1.1526 | 0.00133571 | NSD only | 6.92E-06 |
| Q80WD1 | Rtn4rl2 | Intragenic | 0.6142 | 9.43E-22 | HSD only | 1.93E-08 |
| Q08775 | Runx2 | Intragenic | 2.4509 | 0.00244906 | NSD only | 3.94E-18 |
| O88453 | Safb | Intragenic | 2.4599 | 0.0000256 | NSD only | 0.00089798 |
| Q9QVY3 | Sar1b | TSS | 2.1842 | 0.00227832 | NSD only | 0.0024346 |
| Q64213 | Sf1 | TSS | 8.0000 | 8.91E-08 | NSD only | 1.81E-05 |
| P97710 | Sirpa | TSS | 2.7357 | 0.00054182 | NSD only | 0.00012587 |
| P97849 | Slc27a1 | TSS | 4.8096 | 0.00145773 | NSD only | 3.07E-34 |
| Q9R1B9 | Slit2 | TSS | 0.0766 | 0.00129358 | HSD only | 0.0014185 |
| Q9WVB4 | Slit3 | TSS | 0.5291 | 0.00237351 | HSD only | 0.00047331 |
| Q8R5A0 | Smyd2 | TSS | 1.8398 | 0.00034256 | NSD only | 0.00033483 |
| O54694 | Sptlc2 | TSS | 2.9568 | 0.0000165 | NSD only | 1.34E-09 |
| P70302 | Stim1 | TSS | 1.9580 | 0.00363412 | NSD only | 0.0024346 |
| P70452 | Stx4 | TSS | 2.9551 | 0.00081459 | NSD only | 4.76E-05 |
| Q7TP47 | Syncrip | TSS | 0.6069 | 0.0000758 | 3.8333 | 0.0017924 |
| P20293 | T | TSS | 2.2258 | 0.00032224 | NSD only | 0.0024346 |
| Q60707 | Tbx2 | Intragenic | 1.2118 | 4.04E-08 | NSD only | 0.00089798 |
| Q99JD2 | Tekt1 | TSS | 2.2677 | 0.0000392 | NSD only | 0.0024346 |
| Q9WTQ8 | Timm23 | TSS | 1.2466 | 1.98E-11 | NSD only | 1.52E-07 |
| Q4V899 | Tmem165 | TSS | 2.3419 | 5.31E-08 | NSD only | 1.55E-18 |
| Q5PQM0 | Tmem168 | TSS | 10.5687 | 2.31E-15 | NSD only | 1.81E-05 |
| Q9DCC8 | Tomm20 | TSS | 2.8097 | 0.00060992 | NSD only | 0.00089798 |
| Q9DCK3 | Tspan4 | Intragenic | 1.5576 | 1.41E-16 | NSD only | 0.00089798 |
| Q4QRB4 | Tubb3 | TSS | 0.3446 | 0.000025 | 6.6000 | 7.19E-22 |
| Q9JLA3 | Uggt1 | TSS | 0.3375 | 0.00052776 | 2.3023 | 3.74E-05 |
| P41542 | Uso1 | TSS | 1.8674 | 0.0004832 | NSD only | 4.15E-16 |
| Q9WV55 | Vapa | TSS | 1.5681 | 0.00110753 | NSD only | 4.76E-12 |
| Q80TY5 | Vps13b | TSS | 4.5077 | 2.98E-16 | NSD only | 7.95E-11 |
| Q80TY5 | Vps13b | Intragenic | 1.3103 | 1.27E-11 | NSD only | 7.95E-11 |
| P22725 | Wnt5a | Intragenic | 1.5016 | 0.0000229 | NSD only | 5.87E-08 |
| Q9CR11 | Yeats4 | TSS | 2.6290 | 0.00035212 | NSD only | 1.02E-06 |
